# Ketogenic diet therapy for pediatric epilepsy is associated with alterations in the human gut microbiome that confer seizure resistance in mice

**DOI:** 10.1016/j.celrep.2023.113521

**Published:** 2023-12-08

**Authors:** Gregory R. Lum, Sung Min Ha, Christine A. Olson, Montgomery Blencowe, Jorge Paramo, Beck Reyes, Joyce H. Matsumoto, Xia Yang, Elaine Y. Hsiao

**Affiliations:** 1 Department of Integrative Biology & Physiology, University of California, Los Angeles, Los Angeles, CA 90095, USA; 2 UCLA Goodman-Luskin Microbiome Center, Vatche and Tamar Manoukian Division of Digestive Diseases, David Geffen School of Medicine, Los Angeles, CA 90095, USA; 3 Department of Pediatrics, Division of Pediatric Neurology, David Geffen School of Medicine, University of California, Los Angeles, Los Angeles, CA 90095, USA; 4 Lead contact

## Abstract

The gut microbiome modulates seizure susceptibility and the anti-seizure effects of the ketogenic diet (KD) in animal models, but whether these relationships translate to KD therapies for human epilepsy is unclear. We find that the clinical KD alters gut microbial function in children with refractory epilepsy. Colonizing mice with KD-associated microbes promotes seizure resistance relative to matched pre-treatment controls. Select metagenomic and metabolomic features, including those related to anaplerosis, fatty acid β-oxidation, and amino acid metabolism, are seen with human KD therapy and preserved upon microbiome transfer to mice. Mice colonized with KD-associated gut microbes exhibit altered hippocampal transcriptomes, including pathways related to ATP synthesis, glutathione metabolism, and oxidative phosphorylation, and are linked to susceptibility genes identified in human epilepsy. Our findings reveal key microbial functions that are altered by KD therapies for pediatric epilepsy and linked to microbiome-induced alterations in brain gene expression and seizure protection in mice.

## In brief

Lum et al. find that ketogenic diets (KDs) alter the gut microbiome in children with drug-resistant epilepsy. Mice colonized with KD-associated microbes exhibit seizure resistance. Human donors and mouse recipients share functional features of the KD-associated microbiome linked to hippocampal transcriptomic signatures, epilepsy susceptibility genes, and seizure protection.

## Graphical Abstract

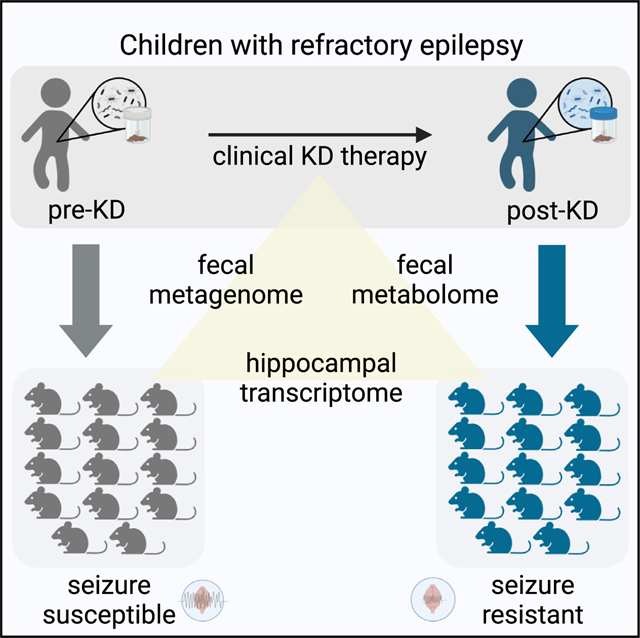

## INTRODUCTION

The low-carbohydrate, high-fat ketogenic diet (KD) is a mainstay treatment for refractory epilepsy, particularly in children who do not respond to existing anti-seizure medications. Its efficacy is supported by multiple retrospective and prospective studies, which estimate that ~30% of pediatric patients become seizure free and ~60% experience substantial benefit with >50% reduction in seizures.^1–4^ However, use of the KD remains low due to difficulties with implementation, dietary compliance, and adverse side effects.^5^ Even with successful seizure reduction, retention on the KD is a reported 13% by the third year of therapy.^6^ Primary reasons for discontinuation include diet restrictiveness and diet side effects, in addition to poor responsiveness. Exactly how the KD confers protection against epilepsy remains unclear, and the biological determinants of patient responsiveness to the KD are poorly understood.

The gut microbiome plays an integral role in mediating effects of diet on host metabolism, neural activity, and behavior.^7,8^ To date, a few clinical studies have reported associations between KD regimens and microbiota alterations in epileptic individuals, with little consistency across reports in the specific microbial taxa or gene pathways involved.^9–11^ We previously reported that KD-induced alterations in the gut microbiome mediate the seizure-protective effects of KD chow in the 6-Hz seizure and *Kcna1* deficiency mouse models for refractory epilepsy.^12^ Similarly, in a rat injury model of infantile spasms, transfer of the KD-induced gut microbiota into naive animals fed control diet (CD) reduced spasms.^13^ Additionally, taxonomic differences in the gut microbiome correlated with KD-induced seizure protection in the *Scn1a* deficiency model for Dravet syndrome.^14^ Together, these findings provide proof of principle that the KD alters the gut microbiome in ways that can promote seizure protection. Whether these results from rodent studies apply to human epilepsy, the human gut microbiome, and clinical KD regimens remains unknown, and the core microbial functions that affect seizure susceptibility are unclear.

We perform a prospective study of KD interventions in children with refractory epilepsy and test causal effects of the human gut microbiome before and after initiating clinical KD regimens on seizure susceptibility in mice. We evaluate microbial functional changes that associate with KD treatment. We identify shared features of the clinical KD-associated gut microbiome that are preserved in recipient mice and correlate with microbiome-dependent seizure protection. Finally, we identify key network interactions between the gut microbiome, metabolites, and brain transcriptome that may contribute to the seizure-protective effects of the KD-associated human gut microbiome.

## RESULTS

### Clinical KD regimens elicit shared functional features of the gut microbiome in a cohort of children with refractory epilepsy

The KD is commonly prescribed for pediatric refractory epilepsy, wherein children consume commercial ketogenic formula and/or fat-rich, carbohydrate-restricted meals with guidance from clinicians and registered dieticians.^5^ KD regimens vary depending on patient tolerability, which dictates the diet ratio of fat to carbohydrate and protein. Additionally, variable food sources determine the specific macro- and micro-nutrients consumed as KD therapy. Moreover, the treatment population varies in genetic risk, seizure semiology, and medication use, among other factors. In order to assess effects of clinically relevant KD treatments for refractory epilepsy on the gut microbiome, we therefore conducted a prospective study of 10 children with pediatric refractory epilepsy newly enrolled in the Ketogenic Diet Program at UCLA Mattel Children’s Hospital (Table S1A). From each patient, we collected a stool sample within 1 day before initiating KD therapy (pre-KD) and after ~1 month of adherence to a clinically guided KD (post-KD), a time point when stabilized microbial responses are expected.^15^

16S rRNA gene sequencing indicated no significant difference in bacterial α diversity in the post-KD fecal microbiota relative to matched pre-KD controls (Figure S1A; Table S2A). There was substantial variation across individuals in baseline microbiota composition (Figure S1B). Additionally, the clinical KD elicited differential shifts in bacterial β diversity across post-KD samples relative to their pre-KD controls, which were not significantly associated with demographic or clinical measures, such as age, sex, and prior anti-seizure medication (Figure S1B; Tables S1A and S1B). Consistent with the inter-individual variation in microbial taxonomic profiles, analysis of composition of microbiomes (ANCOM) and ANOVA analyses identified no significant differences when considering all post-KD samples relative to pre-KD controls (Figures S1C). These results indicate that, within this particular cohort, there are no shared effects of the clinical KD on the microbial composition of the gut microbiota of children with refractory epilepsy.

Functional redundancy is common across microbial species of the human gut.^16^ Given the varied bacterial taxonomic profiles at baseline and in response to dietary treatment, we next asked whether the clinical KD is associated with shared alterations in the functional potential of the gut microbiota. Compared to pre-KD controls, post-KD samples shared a significant decrease in representation of microbial genes belonging to the top 26 most abundant functional pathways, which together comprised >94% of the pathway diversity detected (Figures S1D and S1E; Table S3A). There were no reported changes in total observed pathways (Figure S1F). This suggests that the clinical KD restricts the membership of microbial taxa that harbor genes related to prevalent functions and/or enriches for microbial taxa that harbor genes related to previously underrepresented functions. Post-KD samples exhibited significant enrichment of genes related to formaldehyde assimilation, guanosine nucleotide degradation, and L-proline biosynthesis, and decreased representation of genes related to aerobactin biosynthesis (Figure S1G; Table S3B, see section “discussion”). There were also modest increases in genes related to GDP-mannose biosynthesis, 2-methylcitrate cycle, and glycol metabolism and degradation, and decreases in genes related to polyamine biosynthesis and biotin biosynthesis (Figure S1G). These data suggest that KD regimens that differ in ratio and specific nutritional composition elicit broad shifts in the functional potential of the gut microbiome that are shared across children with varied subtypes of refractory epilepsy.

### Transferring the fecal microbiota from KD-treated pediatric epilepsy patients to mice confers seizure resistance

**Figure 1.**
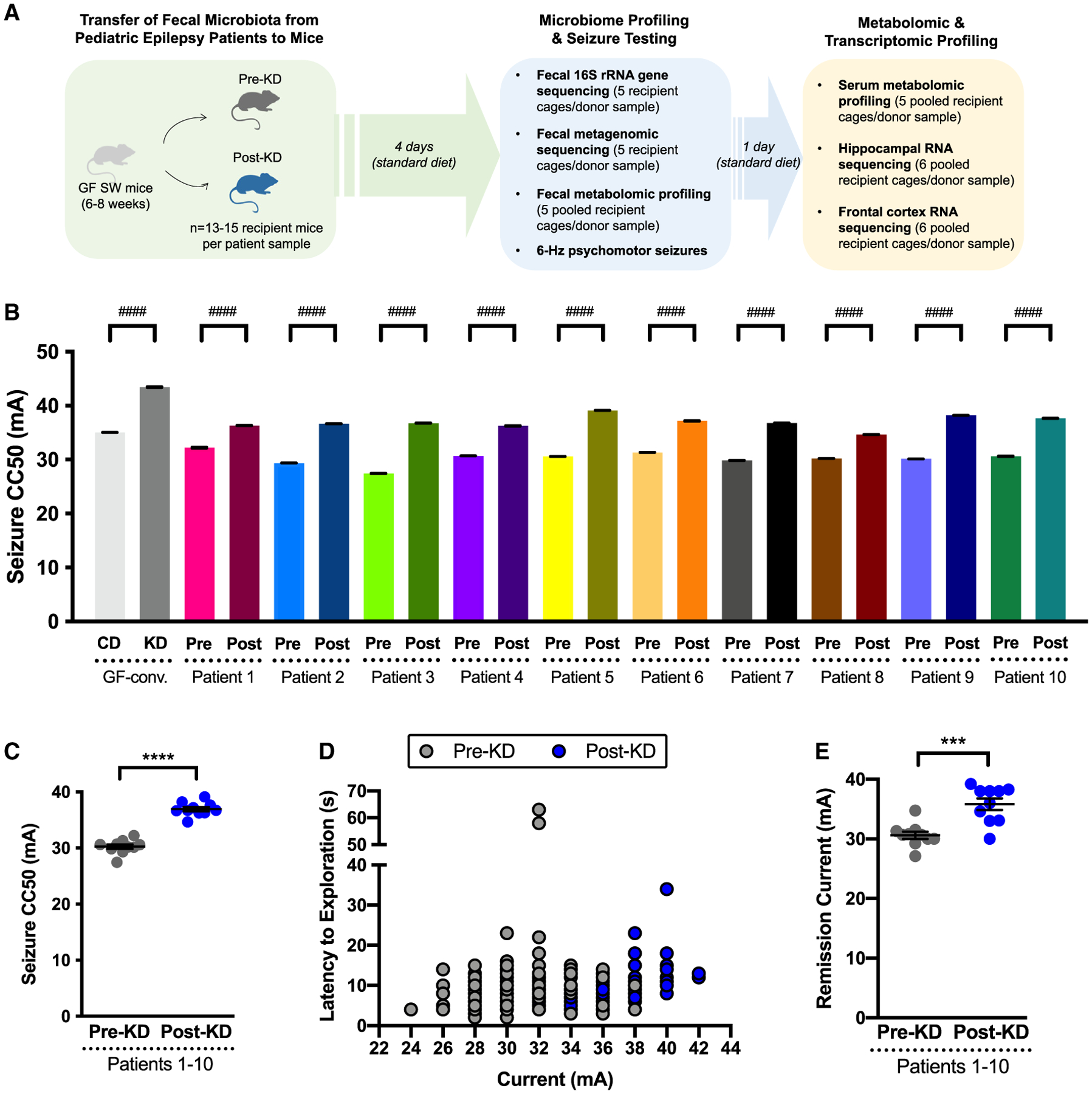
Transfer of the clinical KD-associated gut microbiota from pediatric epilepsy patients to mice confers resistance to 6-Hz seizures (A and B) (A) Experimental schematic and (B) 6-Hz seizure thresholds for mice inoculated with pre-KD and post-KD human microbiota (one-way ANOVA with Tukey’s, n = 13–15 mice/patient sample). (C) Average seizure thresholds of recipient mice per patient sample (two-tailed, unpaired Welch’s t test. n = 10 patients/group). (D) Latency to exploration for all pre-KD (n = 140) and post-KD (n = 141) recipient mice. (E) Average current for remission seizures (two-tailed, unpaired Welch’s t test. n = 10 patients/group). Data are displayed as mean ± SEM. ***p < 0.001, ****p < 0.0001, ^####^p < 0.0001 (within-patient mouse recipients).

Causal influences of the human microbiome can be studied by transferring microbes in a clinical sample into microbiome-deficient mice. To evaluate whether gut microbes associated with the clinical KD affect seizure susceptibility, we inoculated individual cohorts of germ-free (GF) mice with pre-KD and post-KD stool samples and maintained them on standard chow (CD). Each human donor sample (pre-KD and post-KD from 10 individuals, as biological replicates) was inoculated into 13–15 GF mice (as technical replicates) to enable cohort-level testing of susceptibility to 6-Hz seizures (Figure 1A). The 6-Hz model involves low-frequency corneal stimulation to induce acute complex focal-onset seizures reminiscent of human limbic epilepsy.^17^ Consistent with refractory epilepsy, the 6-Hz model is resistant to several anti-seizure medications, but treatment with KD chow protects against seizures in rodents,^18^ raising the intensity of current required to elicit a seizure in 50% of the subjects tested (CC50, seizure threshold). The 4-day time point was chosen as the maximum duration of time that a KD-induced microbiota could be maintained in mice fed CD.^12^

We observed that GF mice colonized with microbes from the post-KD microbiota required greater intensity of current to induce 6-Hz seizures (Figure 1B; Table S4) as compared to controls colonized with pre-KD microbiota. This effect was seen when comparing post-KD vs. pre-KD microbiota transfer for individual technical replicates per patient (Figure 1B) and when data were averaged across all patients (Figures 1C and 1D). Similar increases were seen by transferring a randomly selected post-KD sample into mice pre-treated with broad-spectrum antibiotics to deplete the microbiome (Figures S2A–S2C), suggesting that the increases in seizure resistance are not dependent upon the GF background of recipient mice. Compared to pre-KD controls, mice colonized with microbes from the post-KD microbiota also required increased intensity of current to elicit one or more recurred seizures observed after the initial stimulus-induced seizure (Figure 1E), indicating that transfer of the post-KD human microbiota promotes resistance to both primary induced seizures and remission seizures. On average, the post-KD samples raised seizure thresholds by 22.4% ± 6.4% relative to matched pre-KD controls (Figures 1C and 1D). This aligns with the average effect size of KD chow on wild-type mice (24.5%,^12^), and the observed 24.0% increase seen in GF mice colonized with a conventional adult mouse microbiota (GF-conv) and fed KD chow, as compared to controls fed CD (Figure 1B). Discrepancies in effect size across patients were largely driven by baseline differences in pre-KD conditions (Figure 1B), suggesting that the comparatively low microbial diversity resulting from cross-host species transfer increases seizure susceptibility. Consistent with this, we previously observed that decreasing microbial diversity via antibiotic treatment reduced 6-Hz seizure threshold. Overall, these results indicate that inoculating mice with the clinical KD-associated human gut microbiota increases 6-Hz seizure threshold to levels similar to those seen with direct consumption of experimental KD chow.

Human microbiota transplantation to mice involves oral inoculation with a human stool suspension, which is composed of microbial biomass as well as undigested food matter and secreted molecules from the host and microbiota. As such, effects seen in response to the transfer procedure could be due to the KD-associated gut microbiota or microbiota-independent dietary or host factors. To gain insight into whether bacteria from the gut microbiota are required for mediating the increases in seizure protection seen with inoculation of the human post-KD microbiota into mice, GF mice inoculated with a randomly selected post-KD donor sample were post-treated with antibiotics to deplete the microbiota or with vehicle as negative control (Figures S2D and S2E). Mice that were inoculated with the post-KD sample and treated with vehicle displayed seizure thresholds that were comparable to those seen previously in recipient mice without the added vehicle treatment (Figures S2F, S2G, and 1B; Table S4). This suggests that the post-KD microbiota-induced increases in seizure resistance were maintained beyond the 4-day time point used in our initial transfer experiments (Figures 1B and 1C), extending out to 12 days post inoculation in vehicle-treated mice fed CD (Figures S2F and S2G). In contrast, depletion of gut bacteria in mice that were colonized with the post-KD microbiota decreased seizure thresholds to levels that were lower than previously seen in pre-KD-colonized controls (Figures S2F, S2G, and 1B). These results indicate that bacterial members of the post-KD microbiota are necessary for mediating the increases in seizure threshold seen in response to transfer of the clinical KD-associated microbiota from a pediatric epilepsy patient into mice.

Administration of microbial metabolites or microbiome-dependent molecules, in lieu of viable microbiota, has been reported to ameliorate symptoms of recurrent *Clostridiodes difficile* infection, inflammatory bowel disease, and multiple sclerosis, among other conditions.^19–21^ To gain insight into whether administration of clinical KD-associated intestinal small molecules is sufficient to confer seizure protection in mice, a post-KD donor sample was sterile filtered and then administered to a cohort of GF recipient mice (Figure S3A) alongside controls that were administered the unfiltered post-KD suspension, as done previously for human microbiota inoculation (Figures S3A and 1B). At 4 days post inoculation, mice that were treated with the post-KD filtrate exhibited lower seizure threshold compared to controls that were treated with the corresponding unfiltered post-KD suspension (Figures S3B and S3C; Table S4). These data indicate that clinical KD-associated small molecules in the post-KD fecal sample from a pediatric epilepsy patient are not sufficient to confer persistent seizure protection in mice.

Orally administered microbial metabolites can be rapidly absorbed and cleared from systemic circulation within a few hours of administration.^22,23^ To further assess whether clinical KD-associated intestinal small molecules, including microbial metabolites, acutely modulate seizure susceptibility, mice were orally gavaged with a sterile-filtered post-KD sample and assessed 2 h later for 6-Hz seizure threshold, rather than 4 days later as in the previous experiments (Figure S3D). Mice treated with post-KD filtrate exhibited significantly increased seizure protection compared to controls treated with pre-KD filtrate (Figures S3E and S3F; Table S4), with seizure thresholds that approached those seen after inoculation of the post-KD suspension (Figures S3E, S3G, and 1B). These data indicate that administration of clinical KD-associated intestinal small molecules can acutely confer seizure protection in mice over short timescales (i.e., 2 h; Figures S3D–S3G), which diminishes by 4 days post treatment (Figures S3A–S3C). Taken together, the results suggest that the clinical KD for pediatric refractory epilepsy is associated with alterations in metabolic activities of the gut microbiota that promote seizure resistance in mice.

While the “humanization” of mice with microbiota from clinical stool samples is a powerful tool for translational microbiome research,^24^ the approach has technical and biological limitations that warrant careful consideration.^25^ While much of the taxonomic and functional diversity of the donor inoculum can be recapitulated in recipient mice,^26^ developmental influences and host-specific selection,^27^ among other factors, preclude full “engraftment” of the human gut microbiota in GF mice.^25^ To evaluate the fidelity of fecal microbiota transplantation from pediatric epilepsy patients to GF mice, we subjected both the donor pre-KD and post-KD stool samples and corresponding recipient mouse fecal pellets collected at 4 days post inoculation (the day of seizure testing) to 16S rRNA gene sequencing (Figures 1A and S4A–S4E; Tables S2A and S2B). We observed a significant reduction in α diversity, with an average decrease of 38% for all mouse recipient microbiota relative to all human donor microbiota (Figure S4C), indicating incomplete transfer or engraftment of the human microbiota in mice. These results align with previous reports of reduced bacterial α diversity in mice inoculated with human microbiota, with estimated decreases of 35%, 38%, and 50%,^28–30^ suggesting that we achieved levels of transfer fidelity that are consistent with those in the field. However, the inability to fully recapitulate the taxonomic diversity of the human gut microbiota from pediatric epilepsy patients in mice draws into question whether the increases in seizure resistance seen in mice inoculated with post-KD microbiota are relevant to the actual clinical condition. We therefore focused subsequent experiments on identifying and evaluating the subset of functional features of the KD-associated human gut microbiome that are recapitulated in recipient mice and the microbiome-dependent alterations in host physiology that correspond with seizure protection in mice.

### Select functional features of the clinical KD-associated human microbiome are recapitulated in colonized recipient mice and correlate with seizure protection

**Figure 2.**
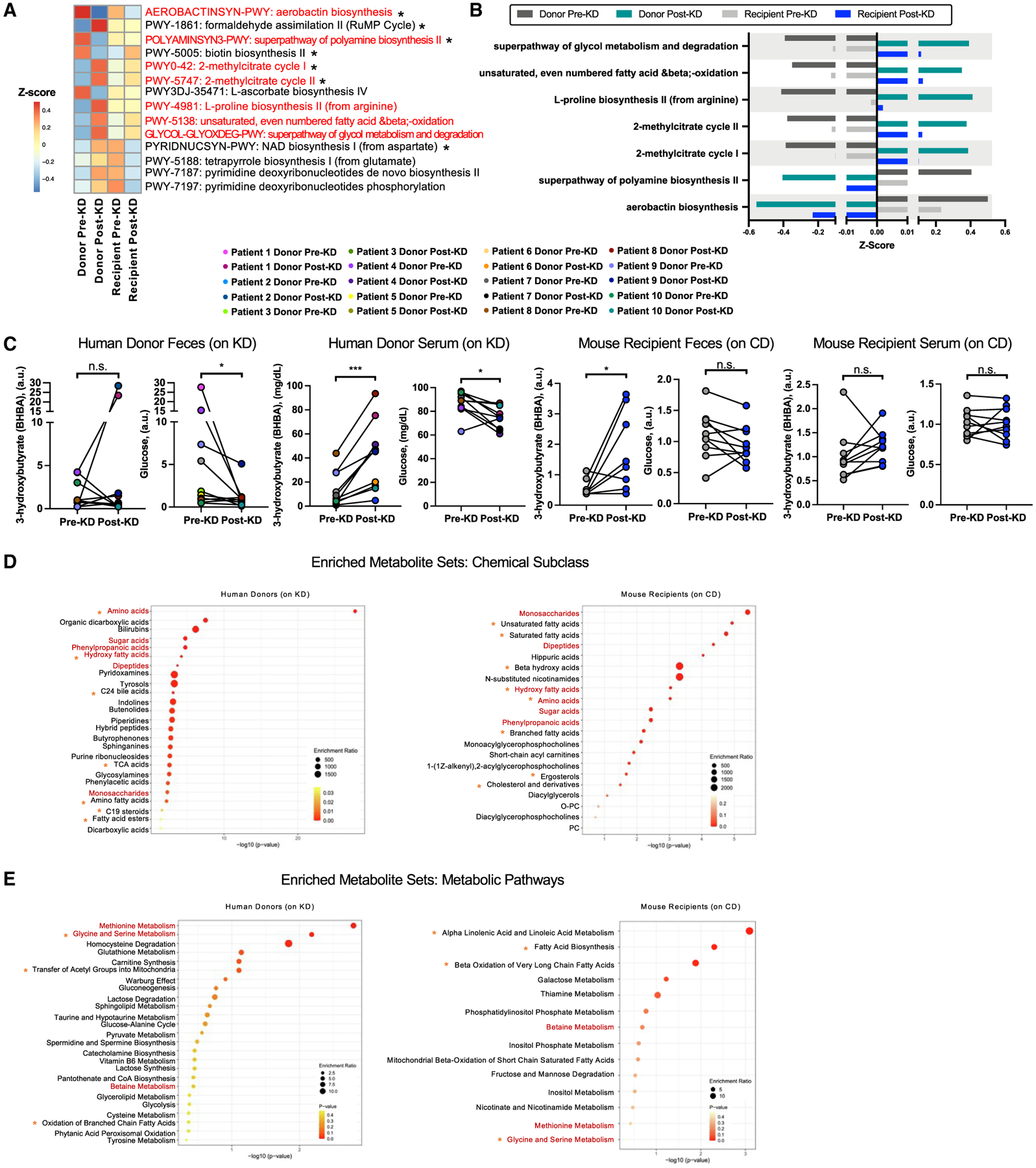
The clinical KD-associated human microbiome exhibits functional alterations that are phenocopied in seizure-protected recipient mice (A) Differentially abundant metagenomic pathways (p < 0.10, where *p < 0.05) in post-KD vs. pre-KD (n = 10/condition; where each recipient n reflects average of five mice per donor sample). Red font indicates changes in same direction in post-KD donors and recipients. (B) Metagenomic pathways differentially abundant in the same direction in post-KD donors and recipients. (C) Beta-hydroxybutyrate and glucose in donor and recipient feces and serum (two-tailed Wilcoxon; n = 10/condition, where each recipient n reflects average of five mice per donor sample). (D) Top 25 enriched chemical subclasses for differentially abundant fecal metabolites (p < 0.05, matched-pairs Student’s t test, n = 10/condition). Red font indicates differential chemical subclasses shared across human and mouse. Orange asterisks indicate additional chemical subclasses relevant to KD based on literature. (E) Top 25 enriched Small Molecule Pathway Database pathways for differentially abundant fecal metabolites (p < 0.05, matched-pairs Student’s t test, n = 10/condition). Data are displayed as mean ± SEM. *p < 0.05; ***p < 0.001; n.s., not statistically significant.

Elucidating how the activity of the gut microbiome is altered by the clinical KD could reveal important insights into its physiological effects. To identify microbiome associations with the clinical KD and further determine which of the associations, if any, may modify seizure risk, we functionally characterized the gut microbiome from pediatric epilepsy patients before and after treatment with the clinical KD, as well as from gnotobiotic mice that were inoculated with the patient samples, and tested for causal outcomes on seizure susceptibility. Metagenomic sequencing and analysis revealed microbial gene pathways that were differentially abundant in post-KD samples relative to pre-KD controls and shared between both human donor and mouse recipient samples (Figure 2A; Tables S3A, S3C, and S3D). Microbial genes relevant to fatty acid β-oxidation, glycol metabolism and degradation, methylcitrate cycle I/II, and proline biosynthesis were similarly elevated in post-KD human samples and post-KD-inoculated mice compared to their pre-KD controls (Figures 2A and 2B; Table S3D). These findings align with reported influences of the KD on fatty acid oxidation,^31^ of carbohydrate restriction on promoting the glyoxylate cycle,^32^ and of fatty acid β-oxidation on the initiation of the methylcitrate cycle.^33^ Proline metabolism involves reactions with glutamine, glutamate, ornithine, and arginine, which might relate to reported effects of KD on amino acid metabolism, particularly of glutamine and glutamate.^34^ In addition, both post-KD human donor and mouse recipient samples exhibited reductions in microbial genes relevant to polyamine biosynthesis and aerobactin biosynthesis (Figures 2A and 2B; Table S3D). Polyamine biosynthesis generates putrescine via the glucogenic amino acid L-arginine, which is consumed in reduced amounts while on the KD. Aerobactin biosynthesis uses the ketogenic amino acid L-lysine, which is also essential for acetyl-coenzyme A (CoA) synthesis and energy production during ketosis. These data suggest that the consumption of a clinical KD by children with refractory epilepsy enriches for gut microbes that have the functional capacity to metabolize dietary fats and to perform anaplerotic reactions when dietary carbohydrates are restricted. The findings indicate that these general features of the KD-associated human gut microbiome are phenocopied in recipient mice that exhibit microbiome-dependent protection against 6-Hz seizures.

The observed metagenomic signatures reveal clinical KD-associated changes in the functional potential of the gut microbiome that are preserved upon transfer to GF mice. To identify clinical KD-induced alterations in the functional activity of the gut microbiome, we examined metabolic profiles in donor fecal samples and of both fecal and serum samples from recipient mice (Tables S5A–S5C). Results from clinical laboratory testing of human blood samples confirmed that the month-long clinical KD regimen elevated serum β-hydroxybutyrate (BHBA) levels and reduced serum glucose levels in pediatric refractory epilepsy patients (Figure 2C). Decreases in glucose, but not BHBA, were similarly seen in stool (Figure 2C), which is consistent with dietary carbohydrate restriction and KD-induced BHBA synthesis by the liver to elevate systemic, but not fecal, BHBA levels.^35^ Transfer of the post-KD human microbiota into mice yielded no significant differences in serum BHBA or glucose relative to pre-KD recipient controls (Figure 2C), indicating that the clinical KD-associated microbiota does not promote key features of ketosis in mice fed the standard CD. However, mice that were inoculated with post-KD human microbiota and fed CD exhibited statistically significant increases in fecal BHBA levels relative to matched pre-KD recipient controls (Figure 2C). This could reflect alterations in intestinal synthesis of BHBA^36^ and/or in microbial utilization of host-derived BHBA^37^ that occur as an artifact of the experimental design. These results suggest that transfer of the clinical KD-associated human gut microbiota into mice promotes resistance to 6-Hz seizures (Figure 1) through mechanisms that act independently of ketosis.

We further performed untargeted metabolomic profiling to identify metabolic patterns that were shared across human donor and mouse recipient samples (Figures 2D, 2E, and S5; Tables S5A, S5B, and S6A). Despite heterogeneity in the patient population and specific clinical KD regimens, 79 metabolites were significantly differentially abundant in fecal samples from post-KD human fecal samples relative to their matched pre-KD controls (Figure S5A; Table S5A). More broadly, 336 of the same metabolites were detected in human fecal samples and mice fed the 6:1 KD chow vs. vitamin- and mineral-matched control chow for 2 weeks, as previously published by our group^12^ (Table S5D). Of these, 35 were differentially abundant in human fecal samples and 169 metabolites in mouse fecal samples (Figure S5B). Twenty were found to be changed in the same direction across human and mouse samples (Figures S5B and S5C). These included KD-induced increases in levels of metabolites related to fatty acid β-oxidation, such as palmitoleoylcarnitine (C16:1) and oleoylcarnitine (C18:1), and a decrease in kynurenine, which have previously been associated with seizure susceptibility.^38^ This statistically significant overlap suggests that there are biochemical changes that are shared across clinical KD treatments for pediatric epilepsy and mouse models of KD and that some of the fecal metabolomic alterations observed in KD-treated epilepsy patients are a direct consequence (rather than correlate) of dietary intervention. Of the 20 significantly differentially abundant metabolites shared in human and mouse, 14 (~70%) were further significantly altered by antibiotic treatment to deplete gut bacteria in KD-fed mice^12^ (Figures S5B–S5D; Table S6B). Altogether, these data indicate that clinical KD regimens alter fecal metabolites in children with refractory epilepsy, a subset of which have the potential to be microbiome dependent.

Although there was substantial variability in composition of microbiota (Figures S1 and S4), fecal samples from mouse recipient cohorts exhibited statistically significant alterations in 45 metabolites that were shared when considering all post-KD samples relative to their pre-KD controls (Figure S5A; Table S5B). However, none of these were identical to the 79 differentially abundant metabolites seen in human donor samples (Figure S5A; Table S5A), which could reflect host-specific metabolite utilization and the fact that recipient mice were fed CD, while human donors were consuming a clinical KD at the time of sample collection. To gain insight into whether the metabolites relate to similar biological functions, we performed metabolite set enrichment analysis (MSEA).^39^ Select chemical classes, including those related to amino acid, hydroxy fatty acid, sugar acid, phenylpropanoic acid, and monosaccharide-related metabolites, were similarly enriched in both human donor and mouse recipient (Figure 2D; Table S6A). Differentially abundant metabolites from human post-KD fecal samples also exhibited enrichment of bile acids and other fatty acid derivatives, which might reflect KD- and/or microbiome-driven alterations in lipid metabolism.^40^

For metabolic pathways, post-KD samples for both human donor and mouse recipient conditions exhibited differential abundance of metabolites related to methionine metabolism, glycine and serine metabolism, and betaine metabolism (Figure 2E; Table S6C). These pathways could reflect known influences of the KD on one-carbon (1C) metabolism, which integrates nutrient availability with cellular nutritional status.^41^ Differentially abundant fecal metabolites from mouse post-KD recipients mapped to pathways related to α-linolenic acid and linoleic acid metabolism, fatty acid biosynthesis, and β-oxidation of very long-chain fatty acids (Figure 2E, right), which aligns with the observed metagenomic enrichment in microbial genes related to fatty acid metabolism (Figures 2A and 2B). Select features in mouse fecal samples were similarly seen in matched mouse serum samples (Tables S5C and S6D); in particular metabolites representing amino acid, hydroxy fatty acid, and unsaturated fatty acid subclasses, and related to α-linolenic acid and linoleic acid metabolism, betaine metabolism, and β-oxidation of fatty acids were altered in both feces and serum of mice receiving post-KD samples relative to pre-KD control samples (Figure S5E; Table S6D). Taken together, these results suggest that the clinical KD induces alterations in the function of the gut microbiome of pediatric epilepsy patients and that a subset of these functional characteristics may be phenocopied upon microbial transfer to mice, which develop microbiome-dependent resistance to 6-Hz seizures.

### Transferring the fecal microbiota from KD-treated pediatric epilepsy patients to mice induces alterations in brain gene expression

**Figure 3.**
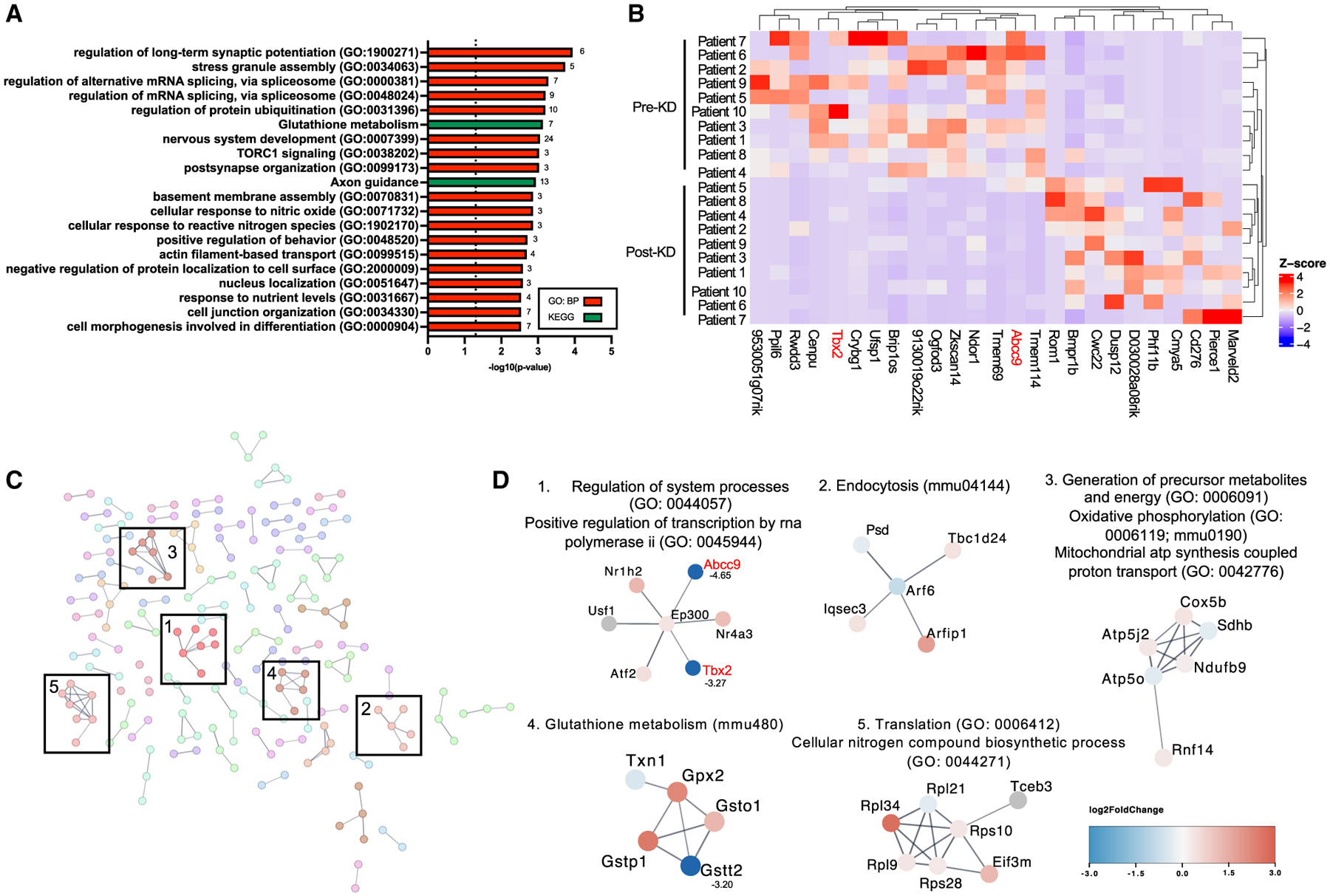
Seizure resistance in mice inoculated with the post-KD microbiota is associated with alterations in the brain transcriptome (A) Biological Process Gene Ontology (GO) of differentially expressed genes (DEGs) (p < 0.05) in post-KD vs. pre-KD mouse hippocampus (n = 10/condition, where each n is pooled from six mice per donor sample). (B) Top 25 DEGs ranked by p value with minimum log2 fold change (log2FC) > 2. log2FC was z score normalized by column. (C) Protein interaction network (enrichment score >0.7) of DEGs that appeared in both GO and STRING network analyses. (D) Functional enrichment of top subnetwork clusters. If log2FC > 3 or <−3, the value is listed next to the node name.

Seizures result from atypical neural function related to discharge of electrical signals or failure to constrain the spread of these signals. To gain insight into how colonization with microbes derived from the fecal microbiota of KD-treated individuals may alter brain function to modify seizure susceptibility, we performed transcriptomic profiling of brain tissues from recipient mice. We focused on the hippocampus and frontal cortex based on their relevance to human epilepsy, their involvement in initiating psychomotor seizures in the 6-Hz seizure assay, and evidence that the microbiome can alter gene expression and metabolites in these brain regions.^42,43^ RNA sequencing of hippocampal tissues revealed differentially expressed genes (DEGs) that were seen in post-KD samples relative to pre-KD controls (Table S7A), including those related to core cell biological processes relating to RNA processing, translation, cellular stress response, TORC1 signaling, regulation of long-term synaptic potentiation, neuronal development, and response to nutrient levels (Figure 3A). The most drastic alterations included upregulation of *Dusp12*, *Bmpr1b*, and *Cmya5* and downregulation of *Abcc9*, *Ufsp1*, and *Tbx2* transcripts (Figure 3B). *Dusp12* is a dual-specificity phosphatase^44^, *Bmpr1b* is a serine/threonine kinase influencing neuronal cell fate,^45^ and *Cmya5* encodes for myospyrn, which is essential for structural integrity during neuritogenesis.^46^ *Abcc9* is an ATP-binding cassette transporter encoding the sulfonylurea receptor 2 subunit for potassium channels,^47^ *Ufsp1* is a Ufm1-specific protease that regulates ubiquitin-like conjugation and has been linked to seizures,^48^ and *Tbx2* is a transcription factor linked to neuronal cell cycle control and neuroinflammation.^49^ Search tool for the retrieval of interacting genes/proteins (STRING) network analysis additionally revealed top protein interaction clusters enriched for essential biological processes including RNA processing, oxidative phosphorylation, and cell cycle regulation, consistent with results from Gene Ontology (GO) enrichment analysis (Figures 3A, 3C, and 3D), as well as endocytosis and glutathione metabolism (Figure 3D).

Some DEGs were also identified in frontal cortical tissues of post-KD recipients relative to pre-KD controls (Table S7B), which, similarly to hippocampus, included those related to core cell biological processes for RNA surveillance and catabolism, cellular stress responses, TORC1 signaling, and further included genes related to potassium ion transport and core carbohydrate metabolism (Figure S6A). The most drastic alterations included upregulation of *Serpinb1a*, *Nqo1*, and *Slc6a12* transcripts and downregulation of *Aldh3b1*, *Setmar*, and *Tfb1m* transcripts (Figure S6B). *Serpinb1a* is a serine/cysteine protease inhibitor,^50^ *Nqo1* encodes an antioxidant enzyme that primarily catalyzes the reduction of quinones,^51^ and *Slc6a12* encodes for a betaine-γ-aminobutyric acid (GABA) transporter.^52^ *Aldh3b1* is an aldehyde dehydrogenase linked to oxidative stress reduction,^53^ *Setmar* encodes a histone-lysine N-methyltransferase,^54^ and *Tfb1m* has been shown to function as methyltransferase.^55^ STRING analysis additionally revealed top protein interaction clusters enriched for transcription regulation, translation, and oxidative phosphorylation, also seen in frontal cortex GO enrichment analysis and in the hippocampal STRING network, as well as clusters enriched for calcium signaling, transcriptional regulation, and translation (Figures S6C and S6D). DEG sets from both hippocampus and frontal cortex were enriched for TORC1 signaling, cellular response to stress, and oxidative phosphorylation through GO enrichment and STRING clustering, which have all been shown to affect seizure susceptibility.^56,57^ The similarities between transcriptomic results from hippocampus and frontal cortex suggest that colonization with post-KD microbes elicits key alterations in host metabolism that affect core biological processes that are generally consistent across different brain regions. Overall, these results indicate that mice that acquire seizure resistance in response to colonization with microbes from the post-KD human gut microbiota exhibit alterations in hippocampal and frontal cortical gene expression, relative to pre-KD recipient controls.

### Multi-omics analysis reveals network connections linking microbial genomic pathways and metabolites to hippocampal transcripts related to epilepsy

**Figure 4.**
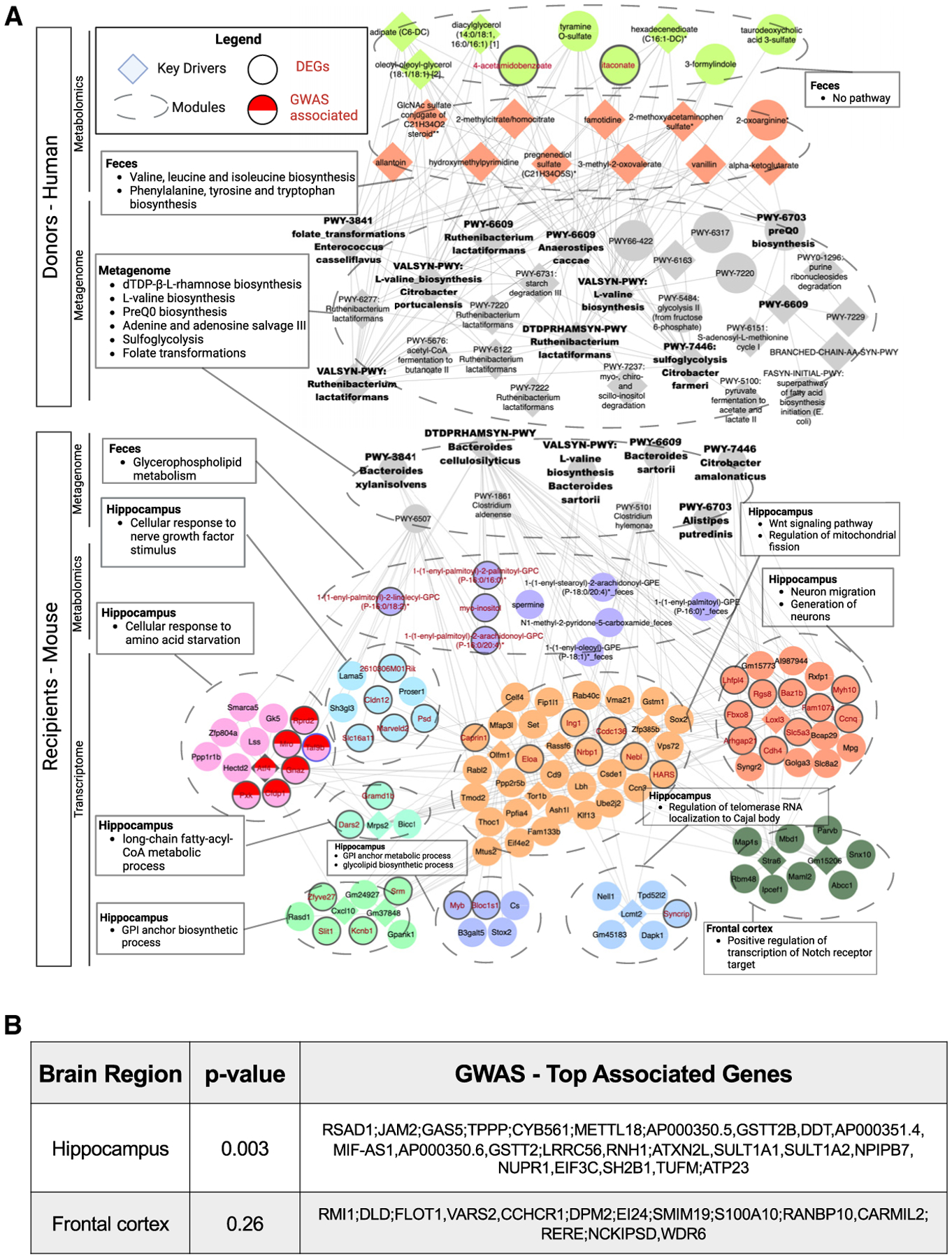
Multi-omic network analysis identifies key microbial genomic pathways and microbially modulated metabolites associated with differential expression of hippocampal transcripts (A) Co-occurrence network and weighted key drivers constructed from human donor fecal metagenomics and metabolomics (top) and mouse recipient fecal metagenomics, fecal metabolomics, serum metabolomics, hippocampal transcriptomics, and frontal cortical transcriptomics datasets (bottom). Red text indicates pathways, metabolites, or genes that were differentially regulated (p < 0.05) in post-KD vs. pre-KD (n = 10/condition, where each recipient n is average of five or six mice). (B) Top epilepsy GWAS genes that map onto mouse hippocampal and frontal cortical DEGs.

To further identify key gut microbial functions that may drive particular brain gene expression signatures, we utilized microbe-metabolite vectors (MMVECs)^58^ to build an integrated network of fecal metagenomic, fecal metabolomic, serum metabolomic, hippocampal transcriptomic, and frontal cortical transcriptomic datasets from recipient mice (Tables S8A and S8B). We generated a parallel network composed of fecal metagenomic and fecal metabolomic datasets from human donors to identify features similarly underscored in both human and mouse networks, suggesting their clinical relevance. The human donor and mouse recipient networks were linked by six common nodes: metagenomic pathways describing adenine and adenosine salvage (PWY-6609), dTDP-L-rhamnose biosynthesis (DTDPRHAMSYN-PWY), folate transformations (PWY-3841), L-valine biosynthesis (VALSYN-PWY), preQ0 biosynthesis (PWY-6703), and sulfoglycolysis (PWY-7446) (Figure 4A, center gray nodes). The shared dTDP-L-rhamnose biosynthesis pathway was identified by weighted key driver analysis in both the human donor and mouse recipient networks, and adenine and adenosine salvage, folate transformations, L-valine biosynthesis, and sulfoglycolysis nodes in the human donor network were further identified by weighted key driver analysis as highly interconnected across the omics datasets and essential regulator nodes of the network^59^ (Figure 4A, gray diamonds). The human donor network also contained additional key driver metagenomic nodes, including acetyl-CoA fermentation to butanoate II (PWY-5676, connected to shared nodes VALSYN-PWY, PWY6609, and DTDPRHAMSYN-PWY), pyruvate fermentation to acetate and lactate II (PWY-5100, connected to shared node PWY-7446), branched-chain amino acid (BCAA) biosynthesis (BRANCHED-CHAIN-AA-SYN-PWY, connected to shared node PWY-6609), and super-pathway of fatty acid biosynthesis (FASYN-INITIAL-PWY, connected to shared node PWY-6609), each of which has previously been linked to the microbiome, KD, and/or neurotransmission.^60–62^ Additionally, the mouse recipient network contained L-isoleucine biosynthesis (PWY-5101), which aligns with the human donor metagenomic node relating to BCAA biosynthesis. The human fecal metabolomic module was also enriched for nodes related to valine, leucine, and isoleucine (BCAAs) biosynthesis and phenylalanine, tyrosine, and tryptophan biosynthesis (Figure 4A, salmon nodes). In the mouse network, the fecal metabolomic module included several nodes related to glycerophospholipid metabolism for fecal metabolites (Figure 4A, purple nodes). Nodes for fecal 1-(1-enyl-palmitoyl)-2-linoleoyl-GPC (P-16:0/18:2)*, 1-(1-enyl-palmitoyl)-2-palmitoyl-GPC (P-16:0/16:0)*, 1-(1-enyl-palmitoyl)-2-arachidonoyl-GPC (P-16:0/20:4)*, and myo-inositol (Figure 4A, red font) were similarly identified as differentially abundant in individual metabolomic analyses for recipient post-KD fecal samples relative to pre-KD controls (Table S5B). The mouse metagenomic modules were linked directly to eight transcriptomic modules for hippocampal genes and indirectly to one frontal cortex module (Figure 4A, bottom, “transcriptome” section). The hippocampal transcript modules were enriched for nodes related to cellular response to nerve growth factor stimulus, Wnt signaling pathway and regulation of mitochondrial fission, neuron migration and generation, cellular response to amino acid starvation, long-chain fatty-acyl-CoA metabolic process, glycosylphosphatidylinositol (GPI) anchor biosynthetic process, GPI anchor metabolic process and glycolipid biosynthetic process, and regulation of telomerase RNA localization to Cajal body. The frontal cortical transcripts were enriched for nodes related to positive regulation of transcription of Notch receptor target (Figure 4A, bottom right). This suggests that these particular biological processes are most closely associated with the microbial functional features identified in the network. The transcript nodes included 38 hippocampal genes that were similarly identified in individual transcriptomic analyses as differentially expressed in post-KD recipient mice relative to pre-KD controls (Figure 4A, red font). The higher direct number of connections between metagenomic modules and hippocampal transcripts suggests that the gut microbiome may exhibit a greater regulatory role for the hippocampus than for the frontal cortex in post-KD recipient mice compared to pre-KD controls. Of particular interest are the indirect links between fecal metabolites related to glycerophospholipid metabolism, which are regulated by the microbiome,^63^ hippocampal transcript modules enriched for cellular response to nerve growth factor stimulus, Wnt signaling, neuron migration, cellular response to amino acid starvation, and GPI anchor biosynthetic and metabolic processes implicated in seizure susceptibility,^64–68^ and the frontal cortical transcript module enriched for transcription of Notch receptor target pathway also implicated in seizure susceptibility.^67,68^

To gain insight into whether the hippocampal and frontal cortical transcripts that co-occur with microbial metagenomic and metabolomic features have been implicated in human epilepsy, single nucleotide polymorphisms identified from epilepsy genome-wide association studies (GWASs) were mapped to genes using hippocampus and frontal cortex splicing quantitative trait loci and expression quantitative trait loci to represent epilepsy-associated genes. The mouse orthologs of these human genes were then compared with hippocampal and frontal cortical transcriptomic results to identify the DEGs in post-KD vs. pre-KD recipients that have been implicated in genetic risk for human epilepsy. There was a statistically significant enrichment of the hippocampal DEGs in the epilepsy GWAS (p = 0.003) but no significant enrichment of the frontal cortical DEGs in the epilepsy GWAS (p = 0.26) (Figure 4B). These results suggest that microbial alterations in hippocampal gene expression may contribute to the microbiome-dependent increases in seizure resistance seen in post-KD recipient mice compared to pre-KD controls. From the co-occurrence network, seven hippocampal DEGs were linked to epilepsy GWAS results: *Atf4*, which encodes a cAMP-response element binding protein; *Taf9b*, which encodes for the 9b subunit of the TFIID transcription initiation factor; *Pxk*, which encodes a serine/threonine kinase that regulates synaptic transmission by binding brain Na-ATPase and K-ATPase; *Rprd2*, which encodes a transcriptional repressor; *Gnaz*, which encodes G protein alpha subunit that regulates ion equilibrium; *Cfdp1*, which encodes a subunit of the chromatin remodeling complex and is important for cell division; and *Mro*, which encodes a nucleolus protein with as-yet unknown function in the nervous system. Overall, the multi-omics analysis of human donor and mouse recipient datasets together with epilepsy GWAS mapping to hippocampal and frontal cortical DEGs identified key microbial genomic pathways and microbially modulated metabolites that may contribute to alterations in the expression of particular hippocampal genes in mice that exhibit microbiome-induced protection against 6-Hz seizures.

## DISCUSSION

This research provides evidence from a treatment study of children with refractory epilepsy, coupled with functional testing in gnotobiotic mice, that clinical KD regimens alter the function of the gut microbiome in ways that could contribute to seizure protection. We assessed microbiome composition and function in 10 children with refractory epilepsy shortly before initiating and approximately 1 month after adherence to classical KD regimens. Following clinical practice, the patient cohort was heterogeneous in type and underlying cause of refractory epilepsy, as well as the ratio of fat to carbohydrate and protein and specific nutritional composition of the KD they consumed (Table S1A). This highlights the diversity of epilepsies that resist current anti-seizure medications and the broad range of KD interventions that are administered to treat pediatric refractory epilepsy. Consistent with this heterogeneity, we observed that participants varied substantially in the composition of the fecal microbiota at baseline and in response to KD treatment. There was no clear KD-induced taxonomic signature of the gut microbiota that was shared across the study population, which contrasts with prior studies of KD treatments for epilepsy that each reported alterations in the gut microbiota in response to a KD. Our results, however, support the finding that little to no consistency in specific microbial taxa affected exists across studies.^69^

Despite variation in composition, we observed evidence of shared functional features of the gut microbiome that were seen with KD treatment across study participants. This aligns with microbial functional redundancy, wherein phylogenetically unrelated species can exhibit the same genetically encoded biological activities.^16^ Microbial genes related to fatty acid β-oxidation, 2-methylcitrate cycle, glycol metabolism, and proline biosynthesis were more highly represented in the gut microbiota of epileptic children after treatment with the KD compared to their internal pre-treatment controls. β-oxidation by select microbes in anaerobic environments enables them to utilize fatty acids from the diet as energy sources, wherein saturated and unsaturated fatty acids are oxidized into acetyl-CoA.^70^ β-oxidation of dietary odd-chain fatty acids additionally produces propionyl-CoA, which can be toxic to cells, so the methylcitrate cycle enables microbes to further catabolize propionyl-CoA into pyruvate and succinate.^71^ Glycol, including glycolate and glyoxylate, metabolism allows microbes to use products from fatty acid oxidation to fuel gluconeogenesis.^72^ Proline synthesis from the central metabolite glutamate, via intermediates amino acids arginine and ornithine, is widely upregulated in bacteria to counteract growth in osmotically unfavorable conditions.^73^ The elevated representation of genes related to these pathways in the post-KD samples suggests that the clinical KD shapes the gut microbiome to enrich microbial taxa that digest fat and synthesize carbohydrates under fat-rich, carbohydrate-limited conditions. These metagenomic features were preserved upon transfer to GF mice that were fed a standard diet, suggesting that the source microbes are maintained under non-ketogenic dietary conditions.

KD therapy induced alterations in lipid and amino acid metabolism, including subsets of amino acids, sugar acids, hydroxy fatty acids, bile acids, and other fatty acid derivatives. In particular, glutamate and ornithine, both precursors of proline, were significantly decreased, which may align with the observed metagenomic alterations in proline biosynthesis pathways. These alterations were similarly seen in mice fed KD and were modified by microbiota depletion, suggesting a causal response to the clinical KD in the human cohort that is dependent on the gut microbiome. Microbially modulated increases in palmitoleoylcarnitine (C16:1) were also seen in KD-fed mice and in post-KD human samples, alongside several other lipid species, aligning with the high fat content of the KD and roles for the gut microbiome in lipid metabolism.^40^

Notably, the individual metabolite changes seen in human donors were not recapitulated by microbiome transfer to GF mice that were fed standard chow. This is perhaps not surprising given the important role of dietary composition in driving microbial activity.^15^ However, a few pathway-level metabolomic changes were consistent between human donors and mouse recipients. Namely, metabolites related to metabolism of methionine, glycine, serine, and betaine were altered across post-KD conditions for human donor and microbiota-recipient mice. Methionine metabolism involves the production of homocysteine, adenosine, cysteine, and α-ketobutyrate, which can then be routed to glucogenic pathways by conversion to propionyl- and succinyl-CoA. Serine, synthesized via glycerate, is used to create glycine (and cysteine) via the homocysteine cycle, which can undergo microbial conversion into pyruvate or glyoxylate. Betaine, derived from diet or synthesized from choline, is metabolized by the gut microbiome^74^ and functions as a methyl donor in transmethylation reactions, including those involved in methionine metabolism. While the relevance to KD and seizure protection is unclear, alterations in peripheral and central amino acid metabolism have been widely implicated in mediating the anti-seizure effects of the KD.^75^ This suggests that transfer of clinical KD-induced gut microbes to mice maintained under non-ketogenic conditions could result in molecular outputs that are distinct from, but functionally similar to, those seen in the donor human sample.

We observed that inoculating mice with human fecal samples collected after clinical KD treatment conferred resistance to 6-Hz seizures across all post-KD donor conditions compared to controls that received the baseline pre-treatment microbiota. There was no correlation with patient responsiveness to diet, as indicated in clinician notes taken at 1 month after adherence to the clinical KD. This may be due to the unreliability of the metric, which was based on parental reporting, as well as the cross-sectional nature of the assessment, given inter-individual variation in latency to respond to KD treatments and the patient’s peak KD ratio. The results highlight the importance of host determinants of KD responsiveness, some of which may mask or block any beneficial influences of the KD-associated microbiota. Many patients included in this study exhibited genetic bases for refractory epilepsy, some of which could be epistatic to functional genomic changes in the KD-associated gut microbiome. Large human studies that subclassify different types of epilepsies and seizure semiologies and comparisons to healthy human controls are warranted to study potential roles for the gut microbiome in modifying or predicting responsiveness to the KD.

Transfer of human KD-associated microbiome to mice revealed microbiota-dependent increases in seizure protection that were associated with brain transcriptomic alterations. Both hippocampus and frontal cortex from recipient mice exhibited enrichment of transcripts related to (1) RNA processing, transcriptional regulation, and translation; (2) TORC1 signaling and cell cycle; and (3) oxidative phosphorylation and cellular stress response when compared to controls colonized with pre-KD microbes. Neuronal excitability requires protein synthesis in response to altered neuronal stimulation, and risk factors for various epilepsies include dysregulation of RNA processing, RNA stability, transcription, and translation.^76^ TORC1 is a major nutrient- and energy-sensing serine/threonine kinase complex that controls cell growth and differentiation by coordinating core processes of transcription, translation, and autophagy. Abnormal regulation of TORC1 signaling has been implicated in many epilepsies and is a therapeutic target.^56^ The KD and select fatty acids inhibit TORC1 activity,^77,78^ suggesting that it may contribute to the anti-seizure effects of the KD. Oxidative phosphorylation is a central process for cellular energy metabolism from nutrients that generates, as a byproduct, reactive oxygen species and is regulated by the retrograde glutamatergic neurotransmitter nitric oxide.^79^ In animal models, it is elevated during seizure activity due to oxidative stress-associated neuronal death,^80^ which can further contribute to epileptogenesis.^57^ The KD reportedly reduces oxidative stress by promoting antioxidant enzymatic activity.^81^ Overall, these results suggest that the KD-associated human gut microbiota alters brain transcriptional pathways that may contribute to protection against 6-Hz seizures in mice.

Integration of multi-omics datasets across human donor and mouse recipients revealed network associations between metagenomic pathways, fecal metabolites, and hippocampal transcripts, suggesting that they may contribute to microbiome-dependent increases in seizure protection. The human and mouse co-occurrence networks were linked by shared metagenomic pathway nodes related to adenine and adenosine salvage, dTDP-L-rhamnose biosynthesis, folate transformations, L-valine biosynthesis, preQ0 biosynthesis, and sulfoglycolysis. Shared key drivers for dTDP-L-rhamnose biosynthesis and nodes for L-valine biosynthesis and adenine and adenosine salvage were linked to hippocampal transcript modules related to neuron generation and migration, cellular response to amino acid starvation, and Wnt signaling and regulation of mitochondrial fission. Catabolism of L-rhamnose produces L-lactaldehyde under aerobic conditions and dihydroxyacetone phosphate under anaerobic conditions; both can be further catabolized to pyruvate, a major precursor for BCAA synthesis and input for the tricarboxylic acid (TCA) cycle.^82,83^ Additionally, in the human donor network, the shared node related to adenine and adenosine salvage was linked to BCAA biosynthesis, while the fecal metabolomic module was enriched for BCAA biosynthesis. BCAAs modulate brain import of precursors required for synthesis of monoamine transmitters.^62,84,85^ BCAAs also serve as nitrogen donors for synthesis of glutamate vs. GABA, and regulate synaptic balance between excitation and inhibition, a key determinant of seizure susceptibility.^61^ Wnt signaling regulates calcium pathways important for hippocampal neurogenesis and is linked to early epileptogenesis.^67^ Additionally, mapping epilepsy risk genes to the co-occurrence network identified seven hippocampal nodes as linked to epilepsy. Of particular interest was *Gnaz*, which encodes G protein alpha-Z, which mediates hippocampal neuronal signal transduction^86^ and is linked to seizure susceptibility.^87^ BCAA derivatives are reported to promote G-protein phosphorylation, and abnormalities in GPCR-mediated neuronal signaling can contribute to seizure susceptibility.^88,89^ Altogether, results from this study reveal that the clinical KD regimens used to treat pediatric refractory epilepsy are associated with alterations in the function of the child microbiome, which causally modify brain function and seizure susceptibility upon transfer to mice. Further research is warranted to define the mechanisms by which the human KD-associated microbiome signals across the gut-brain axis to modify seizure risk and to further assess the potential for identifying microbiome-based interventions that could increase the efficacy of KD treatment, alleviate dietary side effects, and/or ease clinical implementation.

### Limitations of the study

Our study design prioritized experimental reproducibility (with 13–15 mice per patient sample) over patient sample size (10 children with epilepsy). By internally controlling microbiota for each patient, we reasoned that we could evaluate KD responses within a small and heterogeneous patient cohort reflective of the etiopathological variation typically seen in refractory epilepsy. This likely contributed to our finding that there was no shared taxonomic response of the gut microbiome to the clinical KD, despite some shared functional features.

We achieved levels of human-to-mouse transplant fidelity analogous to those reported in the literature even when mice were on a conventional diet rather than KD.^25,26,90^ However, the discrepancies between recipient and donor microbiota draw into question the relevance of findings in gnotobiotic mice to the human condition. To help mitigate this, we focused entirely on features that were differential between post-KD and pre-KD conditions and shared between human donors and mouse recipients. However, we acknowledge that artifacts of the microbiota transfer approach may contribute to the differences observed in the mouse experiments. Nevertheless, the observed results provide important proof of principle that differences in the function of the gut microbiota regulate seizure susceptibility.

We made the major assumption that there exists a singular microbiome-dependent mechanism to increase seizure threshold that is common across all post-KD mouse recipient cohorts relative to all pre-KD controls. Our analysis does not take into account the possibility that there are multiple microbiome-dependent mechanisms that are distinct and that each result in resistance to 6-Hz seizures. Expanded studies that involve sub-classification of the human participants and/or mouse recipients would aid in addressing this prospect.

In light of the patient heterogeneity, small sample size, variable KD regimens, limitations of the microbiota transplantation approach, cross-species and diet comparisons (i.e., human on KD, mouse on standard diet), and assumptions for data analysis, our omics analyses were performed with lenient statistical thresholds for differential abundance (non-adjusted p < 0.05) with a focus on pathway-level signatures. We detected consistent KD-dependent alterations in microbial genes and metabolites in epileptic children undergoing dietary treatment and observed KD- and microbiome-dependent alterations that were shared across human donor and microbiome-recipient mice when using these parameters. The results extend existing pre-clinical research to provide evidence that clinical KD treatments shape the function of the gut microbiome of children with refractory epilepsy in ways that have the potential to causally modify seizure susceptibility.

## STAR★METHODS

### RESOURCE AVAILABILITY

#### Lead contact

Further information and requests for resources and reagents should be directed to and will be fulfilled by the Lead Contact, Elaine Hsiao (ehsiao@g.ucla.edu).

#### Materials availability

This study did not generate new unique reagents.

#### Data and code availability

- Data from 16S rRNA gene sequencing, metagenomic profiling, and associated metadata are presented in Tables S2A, S2B, S3A, and S3C are available online through the NCBI Sequence Read Archive (SRA) repository at SRA: PRJNA1032744. Metabolomic data are presented in Tables S5A–S5D and are available online through Mendeley data: https://doi.org/10.17632/djzyzdbz3z.1. Transcriptomic data are presented in Tables S7A and S7B and available online through Gene Expression Omnibus (GEO) repository with the identification number GEO: GSE225682. Supplemental raw data from Figures 1, 2, 3, 4, and S1–S6 are uploaded to Mendeley Data: https://doi.org/10.17632/5jnk32tfbc.1.
- DOIs are listed in the key resources table and all original code has been deposited at: GitHub: https://github.com/smha118/keto_diet_pediatric_epilepsy and version of record at Zenodo: https://doi.org/10.5281/zenodo.10059754.
- Any additional information required to reanalyze the data reported in this paper is available from the lead contact upon request.

### EXPERIMENTAL MODELS AND STUDY PARTICIPANT DETAILS

#### Human subjects

This study was approved by UCLA’s Institutional Review Board (IRB protocol #15–000453).

Pediatric refractory epilepsy patients were screened and enrolled in collaboration with the Ketogenic Diet Program at UCLA Mattel Children’s Hospital. Prospective participants who met study criteria were provided information detailing this study by phone and email 1–2 weeks before their pre-diet initiation visit. Prior to enrollment, informed signed consent was provided by all participants and their guardians to the program clinical coordinator during the pre-diet initiation appointment. Subjects were enrolled across diverse seizure semiology and prior medical histories. Inclusion criteria: enrolled in UCLA’s program for classical 4:1 KD, children aged 1–10 with refractory epilepsy, any gender, any ethnicity, any previous exposure to AEDs, any seizure semiology. Exclusion criteria: use of antibiotics or probiotics within 4 weeks prior to enrollment, existing diagnosis of gastrointestinal, immunological, or metabolic disorder. Human donor stool samples were collected from 10 participants, each providing 2 stool samples. The first sample was collected within 1 day before starting KD treatment (pre-KD) and the second sample was collected after maintaining on the clinical KD for 1 month (post-KD). Clinical metadata from the medical record were coded and stripped of identifiers before being shared, and included participant demographic data, medical history, AED exposure history, additional medications take during this study, laboratory blood glucose and bloody ketone body levels, seizure severity, seizure frequency, seizure semiology, and dietary regimen (Table S1A).

#### Human stoop sample collection

For in-patient fecal sample collection, once a study participant was admitted to the hospital during the pre-diet initiation visit, they were given a coded stool collection kit and sterile specimen container. Stool samples were freshly collected within 1 day prior to starting the clinical KD treatment (pre-KD). Fresh stool samples were immediately placed on dry ice for short term storage and transportation and were freshly frozen at −80°C for long-term storage. Post-KD stool samples were collected in the same manner as stated above when the study participant returned for the 1-month follow-up visit. For out-patient collection of the post-KD stool sample, which was necessitated because of hospital pandemic policies, a deidentified stool sample collection kit and sterile specimen cup was provided to the patient and guardian along with a pre-labeled return shipping box. After 1 month of the clinical KD treatment, stool samples were collected in a sterile specimen cup, immediately placed in an at home freezer, and the next day either (1) shipped back overnight to UCLA on dry ice or (2) brought with the patient to their 1 month follow-up appointment. Fresh frozen fecal samples were homogenized under liquid nitrogen and 3–500 mg aliquots were made per sample by sterile storage in anaerobic Balch tubes to be used for transplantation, metagenomic, and metabolomic studies.

#### Mice

6–8 week old wild-type germ-free Swiss Webster female mice (Taconic Farms), were bred in UCLA’s Center for Health Sciences Barrier Facility. Breeding animals were fed “breeder” chow (Lab Diets 5K52). Experimental animals were fed vitamin- and mineral-matched control diet (Harlan Teklad TD.150300). Juvenile mice were used to mimic the age range of the human donor population (<10 years old). All animal experiments were approved by the UCLA Animal Care and Use Committee.

### METHOD DETAILS

#### 16S rRNA gene sequencing and analysis

Bacterial genomic DNA was extracted from human or mouse fecal samples using the Qiagen PowerSoil Kit. For human samples, the n reflects one donor sample. For mouse samples, the n reflects independent cages containing 2–3 mice per cage to preclude effects of co-housing on microbiota composition. The sequencing library was generated in line with Caproso et al., 2011.^110^ PCR amplification, run in triplicate, of the V4 region of the 16S rRNA gene was completed using individually barcoded universal primers and 30 ng of the extracted genomic DNA. The PCR product triplicates were pooled and purified using the Qiaquick PCR purification kit (Qiagen). Samples were sequenced using the Illumina MiSeq platform and 2 × 250bp reagent kit for paired-end sequencing at Laragen, Inc. Amplicon sequence variants (ASVs) were chosen by closed reference clustering based on 99% sequence similarity to the SILVA138 database. Taxonomy assignment, rarefaction, and differential abundance testing were performed using QIIME2 2022.2.^92,94^ ANCOM analysis were performed using feature table outputs generated from QIIME2. Feature tables were prepared using standard methodology recommended by QIIME2 implementing add-pseudocount imputation to address all zero-values followed by a log-transformation before standard ANCOM analysis.^92^ Multivariate association analysis of collected donor patient clinical metadata was performed using MaAslin2 with recommended and appropriate normalization (total sum squares), transformation (log transformation), and analysis model (linear model) parameters.^102^

#### Fecal shotgun metagenomics

Bacterial genomic DNA was extracted from human or mouse fecal samples using the Qiagen PowerSoil Kit. 1 ng of DNA was used to prepare DNA libraries using the Nextera XT DNA Library Preparation Kit (Illumina) and genomic DNA was fragmented with Illumina Nextera XT fragmentation enzyme. IDT Unique Dual Indexes were added to each sample before 12 cycles of PCR amplification. AMpure magnetic Beads (Beckman Coulter) were used to purify DNA libraries which were eluted in QIAGEN EB buffer. Qubit 4 fluorometer and Qubit dsDNA HS Assay Kit were used for DNA library quantification. Libraries were then sequenced on Illumina HiSeq 4000 platform 2×150bp at a 6M read depth using by CosmosID. Metagenomic data was analyzed using HUMAnN 3.0^101^ and MetaCyc database to profile gene families and pathway abundance. File2meco R package was used for MetaCyc pathway hierarchical classification.^103^ MaAsLin 2.0^102^ was used to assess significant pathway associations between pre-KD and post-KD with a non-adjusted p value cutoff of 0.1, where non-adjusted p value <0.05 pathways are indicated in the figure by asterisk. Heatmaps were generated using the pheatmap v1.0.12 package for R.

#### Human donor fecal microbiota transfer

To prepare collected human stool samples for transplantation studies, the frozen stool sample was pulverized into a powder under liquid nitrogen stream in a sterile heavy-duty foil covered mortar and pestle, aliquoted at 500mg per tube into 2mL screw cap tubes, and frozen at −80C.

A single 500 mg aliquot of human stool sample was entered into a Coy anaerobic chamber and resuspended in pre-reduced 1× PBS +0.05% L-cysteine. The sample was homogenized using sterile borosilicate glass beads and passed through a 100um filter. GF Swiss Webster mice were colonized by oral gavage with 200uL fecal suspension. Excess fecal suspension was resuspended and stored at −80C in pre-reduced 1× PBS +0.05% L-cysteine +15% glycerol. For administration of fecal filtrates, the fecal suspension was passed through a sterile 0.2 μm filter before colonization via oral gavage using 200uL fecal filtrate.

#### 6-Hz psychomotor seizure assay

6-Hz psychomotor seizure assay testing was conducted following Samala et al.^111^ One drop (~50 μl) of 0.5% tetracaine hydrochloride ophthalmic solution was applied to the corneas of each mouse 15 min before stimulation. A thin layer of electrode gel (Parker Signagel) was applied directly to the corneal electrodes and was reapplied before each trial. A constant-current current device (ECT Unit 57800, Ugo Basile) was used to deliver current through the corneal electrodes at 3s duration, 0.2 ms pulse-width and 6 pulses/s frequency. CC50 (the milliamp intensity of current required to elicit seizures in 50% of the mouse cohort) was measured as a metric for seizure susceptibility. Pilot experiments were conducted to identify 28 mA as the CC50 for SPF wild-type Swiss Webster mice, aged 6–8 weeks. Each mouse was seizure-tested only once, and thus at least n > 13 mice were used to adequately power each cohort. To determine CC50s for each tested cohort, 28 mA of current was administered to the first mouse per cohort, followed by stepwise fixed increases or decreases by 2 mA intervals. Mice were restrained manually during stimulation and then released into a new cage for behavioral observation. Quantitative measures for falling, tail dorsiflexion (Straub tail), forelimb clonus, eye/vibrissae twitching, and behavioral remission were scored manually. For each behavioral parameter, we observed no correlation between percentage incidence during 28+ mA seizures between pre-KD or post-KD microbiota status, suggesting a primary effect of the microbiota on seizure incidence rather than presentation or form. Latency to exploration (time elapsed from when an experimental mouse is released into the observation cage (after corneal stimulation) to its first lateral movement) was scored manually with an electronic timer. Mice were blindly scored as protected from seizures if they did not show seizure behavior and resumed normal exploratory behavior within 10 s. Seizure threshold (CC50) was determined as previously described,^112^ using the average log interval of current steps per experimental group, where sample n is defined as the subset of animals displaying the less frequent seizure behavior. Data used to calculate CC50 are also displayed as latency to explore for each current intensity, where n represents the total number of biological replicates per group regardless of seizure outcome.

#### Antibiotic treatment

Transplanted mice were gavaged with a solution of vancomycin (50 mg/kg), neomycin (100 mg/kg) and metronidazole (100 mg/kg) every 12 h daily for duration of antibiotic treatment.^113^ Ampicillin (1 mg/mL) was provided *ad libitum* in drinking water. For mock treatment, mice were gavaged with a similar volume of 1x PBS (vehicle) water every 12 h daily for 5 days. Antibiotic-treated mice were maintained in sterile caging with sterile food and water and handled aseptically for the remainder of the experiments. Fecal pellets were collected daily in sterile tubes and DNA was isolated using the QIAamp Fast DNA Stool Mini Kit. Quantitative real-time PCR analysis was performed in 20uL reactions run in triplicate using a QuantStudio5 instrument (Applied Biosystems) with SYBR Green and forward primer UniF340 and reverse primer UniR514 under the following PCR protocol: 95°C for 10 minutes; 40 cycles of 10 seconds at 95°C and 45 seconds at 62°C. Melt curves and Ct values were assessed. Please also see Figure S2E.

#### Fecal and serum metabolomics

Previously collected human donor fecal samples were aliquoted as described in section “human donor fecal microbiota transfer”. Mouse fecal samples were collected from mice housed across independent cages, with four cages housing 3 mice and one cage housing 2 mice. Mouse serum samples were collected by cardiac puncture and separated using SST vacutainer tubes, then frozen at −80C. Samples were prepared using the automated MicroLab STAR system (Hamilton Company) and analyzed on GC/MS, LC/MS and LC/MS/MS platforms by Metabolon, Inc. Protein fractions were removed by serial extractions with organic aqueous solvents, concentrated using a TurboVap system (Zymark) and vacuum dried. For LC/MS and LC-MS/MS, samples were reconstituted in acidic or basic LC-compatible solvents containing >11 injection standards and run on a Waters ACQUITY UPLC and Thermo-Finnigan LTQ mass spectrometer, with a linear ion-trap frontend and a Fourier transform ion cyclotron resonance mass spectrometer back-end. For GC/MS, samples were derivatized under dried nitrogen using bistrimethyl-silyl-trifluoroacetamide and analyzed on a Thermo-Finnigan Trace DSQ fast-scanning single-quadrupole mass spectrometer using electron impact ionization. Chemical entities were identified by comparison to metabolomic library entries of purified standards. Following log transformation and imputation with minimum observed values for each compound, post-KD vs. pre-KD comparisons for human fecal, and mouse serum and fecal data were analyzed by paired t test. Metabolomic data from SPF or antibiotic-treated mice fed KD vs. CD chow were acquired from Olson et al., 2018, as log transformed and imputed with minimum observed values for each compound. Data were analyzed using two-way ANOVA to test for group effects. P and q-values were calculated based on two-way ANOVA contrasts. Principal components analysis was used to visualize variance distributions. Supervised Random Forest analysis was conducted to identify metabolomics prediction accuracies. Metabolite set enrichment analysis (MSEA) using the Metaboanalyst 5.0 platform^39^ was performed on human fecal, mouse fecal, and mouse serum metabolites statistically significantly altered in post-KD compared to pre-KD (non-adjusted p value <0.05). Metabolite sets were analyzed for chemical sub-class enrichment and metabolite pathway enrichment, using The Small Molecule Pathway Database (SMPDB).

#### Transcriptomics

Recipient mice were sacrificed on day 4 post-colonization. Hippocampus and frontal cortex were dissected from pre-KD and post-KD recipient mice (n = 6 per cohort) and immediately placed in Trizol. RNA was extracted using the PureLink RNA Mini kit with Turbo DNAse treatment. RNA was prepared using the TruSeq RNA Library Prep kit and 2Å ~ 69-bp paired-end sequencing was performed using the Illumina HiSeq 4000 platform by the UCLA Neuroscience Genomics Core. FastQC v0.11.5, bbduk v35.92, and RSeQC v2.6.4 were used for quality filtering, trimming, and mapping. Reads were aligned to UCSC Genome Browser assembly ID: mm10 using STAR v2.5.2a, indexed using samtools v1.3, and aligned using HTSeq-count v0.6.0. Differential expression analysis was conducted using DESeq2 v1.24.041. Heatmaps were generated using the pheatmap v1.0.12 package for R. GO term enrichment analysis of differentially expressed genes with non-adjuated p value <0.05 was conducted using enrichR v3.1. Protein interaction networks were generated using STRING v10.5. Functional enrichments of network nodes were categorized by GO: biological process, molecular function, and cellular component.

#### Multi-omics integration

To assess the relationships across omics layers, we first carried out dimension reduction for each data set using weighted gene co-expression network analysis (WGCNA v1.72.1)^109^ Metabolomics for human donors and mouse recipients and RNA-seq for mouse recipients (hippocampus and frontal cortex) were used to build WGCNA modules within each dataset, where modules represent clusters of highly co-regulated/expressed molecules which are typically involved in similar biological functions. For metabolomics data, *goodSamplesGenes* function was first used with default parameters to filter out sparse metabolites across samples before constructing networks; this step was not used for RNAseq data. Standard WGCNA steps were then carried out for the filtered metabolomics and RNAseq data. Module eigengenes (MEs), or the first axis of principal component were calculated from each module. MEs were then targeted for correlation analysis with the metadata (pre-KD vs. post-KD and responder vs. non-responder). Modules that had significant correlation (p-val <0.05) with the metadata were chosen for subsequent integrative analysis.

A systematic network that combined all omics data was inferred based on the probability of co-occurrence (POC) between molecules from different omics data. To calculate POC, we leveraged a neural-net based tool called MMVEC v1.0.6 with default parameters.^58^ The subset of raw data that contains module components that were selected from WGCNA analysis were log normalized and combined based on sample ID. This combined data matrix was then used as input for MMVEC. For example, on donor side, modules from fecal metagenome and metabolomics were added together and, on the recipient side, the combined matrix contained the raw data from metagenome, metabolomics, and RNAseq. Due to high density of the overall network generated from MMVEC, the top 10% of POC connections were retrieved to minimize overall complexity of the network for both donors and recipients using in-house python script (https://github.com/smha118/keto_diet_pediatric_epilepsy).

The networks of modules from individual omics layers comprising of pre-KD (n = 10) and post-KD (n = 10) donor metagenome and metabolomic samples and recipient pre-KD (n = 10, where each n reflects average of 5 technical replicate recipient mice per donor patient sample]) and post-KD (n = 10, where each n reflects average of 5 technical replicate recipient mice per donor patient sample]) metagenome, pre-KD (n = 10, where each sample is pooled from 5 recipient mice per donor patient sample) and post-KD (n = 10, where each sample is pooled from 5 recipient mice per donor patient sample) metabolomics, and pre-KD (n = 10, where each sample is pooled from 6 recipient mice per donor patient sample) and post-KD RNAseq (n = 10, where each sample is pooled from 6 recipient mice per donor patient sample), as well as differentially expressed/abundance molecules were then seeded into Mergeomics v3.16 pipeline along with the integrated network generated with MMVEC for weighted key driver analysis (wKDA) to identify key drivers of the networks.^59^ wKDA uses a χ^2^ -like statistic to identify molecules that are connected to significant larger module components than what would be expected by random chance. The analysis was done on the human and mouse networks separately. To further look into the network that are relevant to ketogenic diet and epilepsy, we selected key drivers (KDs) based on i) the number of modules that a key driver was invoked related to, ii) their relation to the Ketogenic diet or epilepsy. A subset of nodes in each module that were connected to the KDs were collected. These nodes were retrieved with the following priorities i) they are part of differentially regulated molecules ii) POC value with KDs. Finally, the network was visualized using Cytoscape.^104^ To minimize overall density of the network, we chose to show the key drivers from Mergeomics with the highest occurrence in their respective MEs and with >5 degrees of connectivity.

#### Marker set enrichment analysis (MSEA) to connect hippocampus and frontal cortex DEGs with epilepsy GWAS

To assess the potential role of the DEGs from the hippocampus and frontal cortex in epilepsy, we collected the summary statistics of the latest epilepsy GWAS.^114^ Single nucleotide polymorphisms (SNPs) that had a linkage disequilibrium of r^2^ > 0.5 were filtered to remove redundancies. To map the epilepsy GWAS SNPs to genes, we used GTEx version 8 eQTL and sQTL data for brain hippocampus and brain frontal cortex,^115^ which help us derive genes likely to be regulated by the SNPs. We next used the MSEA function of the Mergeomics package^59^ to compare epilepsy disease association p values of the SNPs representing the DEGs (hippocampus or frontal cortex) with those of the SNPs mapped to random genes to assess whether the DEGs contain SNPs that show stronger epilepsy association than random genes using a χ^2^-like statistic.

### QUANTIFICATION AND STATISTICAL ANALYSIS

Statistical analyses were conducted using Prism8 software v8.2.1 (GraphPad). Before statistical analysis, data was assessed for distribution to determine appropriate statistical tests to use. Data were plotted in figures as mean ± SEM. For figures: 1B, S2B, S2F, S3B, and S3E *n* = the number of technical replicates. For all other figures, *n* = the number of biological replicates. No samples or animals were excluded from data analysis. Differences between two sample conditions from parametric data sets were analyzed using two-tailed, paired Student’s t-test. Differences between two sample conditions from nonparametric data sets were analyzed using two-tailed, Wilcoxon matched-pairs signed rank test. For differences among >2 groups when analyzing one variable, a one-way ANOVA with Tukey’s post hoc test was used. For differences among ≥2 groups with two variables, a two-way ANOVA with Sidak’s post hoc test was used. For technical replicates from within-patient analysis (Figures: 1B, S2B, S2F, S3B, and S3E), differences from the above tests are denoted by: ^#^p < 0.05; ^##^p < 0.01; ^###^p < 0.001; ^####^p < 0.0001. For biological replicates (all other figures), differences from the above tests are denoted by: *p < 0.05; **p < 0.01; ***p < 0.001; ****p < 0.0001. Non-significant differences are denoted in the figures using “n.s”.

## Supplementary Material

1

2

3

4

5

6

7

8

9

10

## Figures and Tables

**Table:** KEY RESOURCES TABLE

REAGENT or RESOURCE	SOURCE	IDENTIFIER
Biological samples		
Stool samples from human subjects	This study	N/A
Chemicals, peptides, and recombinant proteins		
Vancomycin hydrochloride	Chem-Impex International	00315
Neomycin trisulfate salt hydrate	Sigma-Aldrich	N1876
Metronidazole	Sigma-Aldrich	M1547
Ampicillin sodium salt	Sigma-Aldrich	A9518
TURBO DNase	Invitrogen	AM2238
Ultrapure water	ThermoFisher	10977015
1x PBS	ThermoFisher	10010023
SYBR Green	ThermoFisher	4344463
Tetracaine Hydrochloride Opthalmic Solution, USP 0.5%	Oceanside Pharmaceuticals	68682-920-64
Critical commercial assays		
DNeasy PowerSoil Kit	Qiagen	12888-50
QIAamp Fast DNA Stool Mini Kit (see Figure S2E)	Qiagen	51604
Qiaquick PCR purification kit	Qiagen	28104
PureLink RNA Mini Kit	Invitrogen	12183018A
QuantSeq FWD’ mRNA-Seq Library Prep Kit	Lexogen	N/A
Deposited data		
16S rRNA gene sequencing	NCBI Sequence Read Archive (SRA)	PRJNA1032744
Metagenomic sequencing	NCBI Sequence Read Archive (SRA)	PRJNA1032744
Untargeted metabolomics	https://data.mendeley.com/	https://doi.org/10.17632/djzyzdbz3z.1
Hippocampal transcriptomics	Gene Expression Omnibus (GEO)	GSE225682
Frontal cortex transcriptomics	Gene Expression Omnibus (GEO)	GSE225682
Multi-omic integration WGCNA modules	GitHub	https://github.com/smha118/keto_diet_pediatric_epilepsy
Multi-omic integration WGCNA modules	Zenodo	https://doi.org/10.5281/zenodo.10059754
Supplemental Raw Data	https://data.mendeley.com/	https://doi.org/10.17632/5jnk32tfbc.1
Experimental models: Organisms/strains		
Swiss Webster	Taconic Farms	TAC-SW
Oligonucleotides		
Forward primer for digital PCR: UN00F2, 5′- CAGCMGCCGCGGTAA-3	Integrated DNA Technologies	N/A
Forward primer for qPCR: UniF340, 5’-ACTCCTACGGGAGGCAGCAGT-3’ (see Figure S2E)	Integrated DNA Technologies	N/A
Reverse primer for qPCR: UniR514, 5’-ATTACCGCGGCTGCTGGC-3’ (see Figure S2E)	Integrated DNA Technologies	N/A
Reverse primer for digital PCR: UN00R0, 5′-GGACTACHVGGGTWTCTAAT-3′ [1, 3])	Integrated DNA Technologies	N/A
Software and algorithms		
Deblur	https://github.com/biocore/deblur	Amir et al.^91^
QIIME2-2022.2	https://qiime2.org/	Bolyen et al.^92^
FastQC v. 0.11.9	https://github.com/s-andrews/FastQC/releases/tag/v0.11.9	Andrews^93^
ANCOM	https://github.com/FrederickHuangLin/ANCOM-Code-Archive	Mandal et al.^94^
Trimmomatic	https://github.com/timflutre/trimmomatic	Bolger et al.^95^
HISAT2	http://daehwankimlab.github.io/hisat2/	Kim et al.^96^
HTSeq-count	https://github.com/htseq/htseq	Anders et al.^97^
DESeq2	https://bioconductor.org/packages/release/bioc/html/DESeq2.html	Love et al.^98^
RStudio 2022.07.2	https://www.r-project.org/	RStudio Team^99^
bioBakery	https://github.com/biobakery/biobakery	McIver et al.^100^
HUMAnN 3.0	https://github.com/biobakery/humann	Beghini et al.^101^
MetaPhlAn 3.0	https://github.com/biobakery/MetaPhlAn	Beghini et al.^101^
MaAsLin 2.0	https://github.com/biobakery/biobakery/wiki/maaslin2	Mallick et al.^102^
file2meco	https://github.com/ChiLiubio/file2meco	Liu et al.^103^
MetaboAnalyst 5.0	https://www.metaboanalyst.ca/home.xhtml	Pang et al.^39^
Cytoscape	https://cytoscape.org/	Shannon et al.^104^
EnrichR	https://maayanlab.cloud/Enrichr/	Chen et al.; Kuleshov et al. and Xie et al.^105–107^
STRING	https://string-db.org/	Szklarczyk et al.^108^
WGCNA	https://horvath.genetics.ucla.edu/html/CoexpressionNetwork/Rpackages/WGCNA/	Langfelder and Horvath^109^
MMVEC	https://github.com/biocore/mmvec	Morton et al.^58^
wKDA	http://mergeomics.research.idre.ucla.edu/	Ding et al.^59^
BlueBee	Lexogen	1864011
Prism software	GraphPad	v 8.2.1
Other		
“Breeder” chow	Lab Diets	5K52
Control diet	Harlan Teklad	TD.150300
4200 Tapestation System	Agilent	G2991AA
QX200 Droplet Generator	Bio-Rad Laboratories	1864002
ECT Unit	Ugo Basile	57800

## Highlights

- Colonizing mice with KD-associated human gut microbes increases seizure resistance
- Human donor and mouse recipients share select metagenomic and metabolomic features
- Mice colonized with KD-associated gut microbes exhibit altered brain transcriptomes
- Multi-omic analysis shows candidate microbial key drivers of seizure protection
