# Supplementary material for: Ketogenic diet therapy for pediatric epilepsy is associated with alterations in the human gut microbiome that confer seizure resistance in mice
Source: Cell Rep. Author manuscript; Available in PMC 2024 Jan 5. (PMC10769314; doi:10.1016/j.celrep.2023.113521)
Supplement: 1 [file NIHMS1954782-supplement-1.pdf]

Cell Reports, Volume *42*

Supplemental information

Ketogenic diet therapy for pediatric epilepsy is associated with alterations in the human gut microbiome that confer seizure resistance in mice

Gregory R. Lum, Sung Min Ha, Christine A. Olson, Montgomery Blencowe, Jorge Paramo, Beck Reyes, Joyce H. Matsumoto, Xia Yang, and Elaine Y. Hsiao

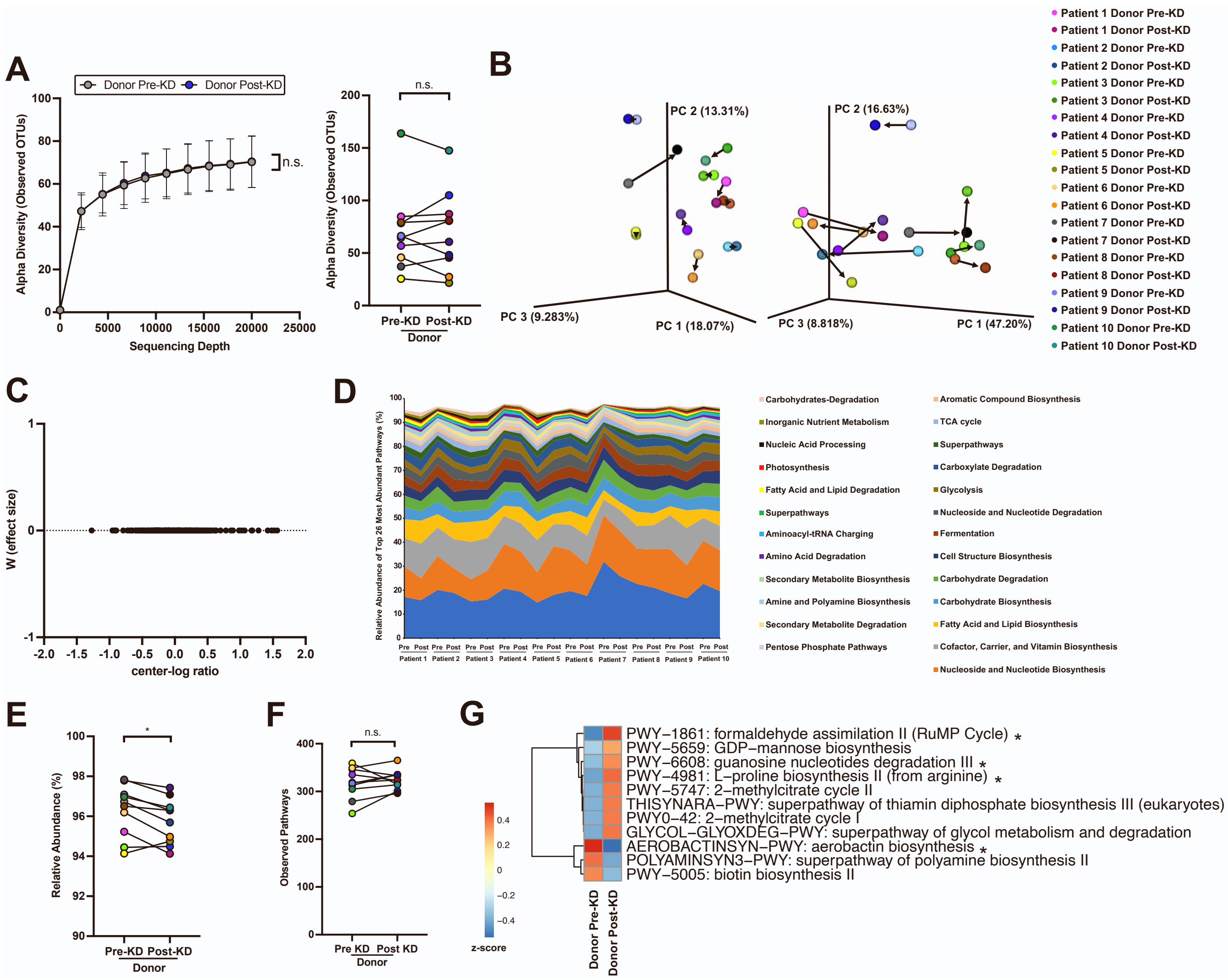

Figure S1: Clinical KD is associated with alterations in the functional potential, but not composition, of the gut microbiota in a cohort of children with refractory epilepsy, Related to Figure 1. **(A)** Alpha diversity as measured by rarefaction curve (left) and observed OTUs (right) of matched donor pre-KD (n=10) and post-KD (n=10) fecal microbiota samples showing no difference in alpha-diversity. (two-way ANOVA with Sidak (left); two-tailed, Wilcoxon matched-pairs signed rank test (right)). **(B)** Principal coordinate analysis of unweighted (left) and weighted (right) UniFrac distances from 16S rRNA gene sequencing of donor pre-KD (n=10) and post-KD (n=10) fecal microbiota samples shifting composition when introduced to the clinical KD. **(C)** ANCOM taxonomic differential abundance testing displaying no differentially abundant taxa by the W score (effect size) metric when comparing donor pre-KD (n=10) and post-KD (n=10). **(D)** Total composition per human donor sample of the top 26 MetaCyc superclass metagenome functional pathways accounting for >94% of relative abundance, for each donor pre-KD (n=10) and post-KD (n=10) fecal microbiota samples. **(E)** Difference in total abundance of the 26 most abundant pathways between matched donor pre-KD (n=10) and post-KD(n=10) fecal microbiota samples (two-tailed, Wilcoxon matched-pairs signed rank test). **(F)** Total number of observed MetaCyc functional pathways in matched donor pre-KD (n=10) and post-KD (n=10) fecal microbiota samples (two-tailed, Wilcoxon matched-pairs signed rank test). **(G)** Heatmap displaying differentially abundant MetaCyc functional pathways associated with donor post-KD (n=10) relative to pre-KD (n=10) by MaAsLin2 analysis with a p-value < 0.1. (pathways with a p-value <0.05 are denoted with *). Data is displayed as mean ± SEM, unless otherwise noted. *p < 0.05, n.s = no statistical significance; KD, ketogenic diet.

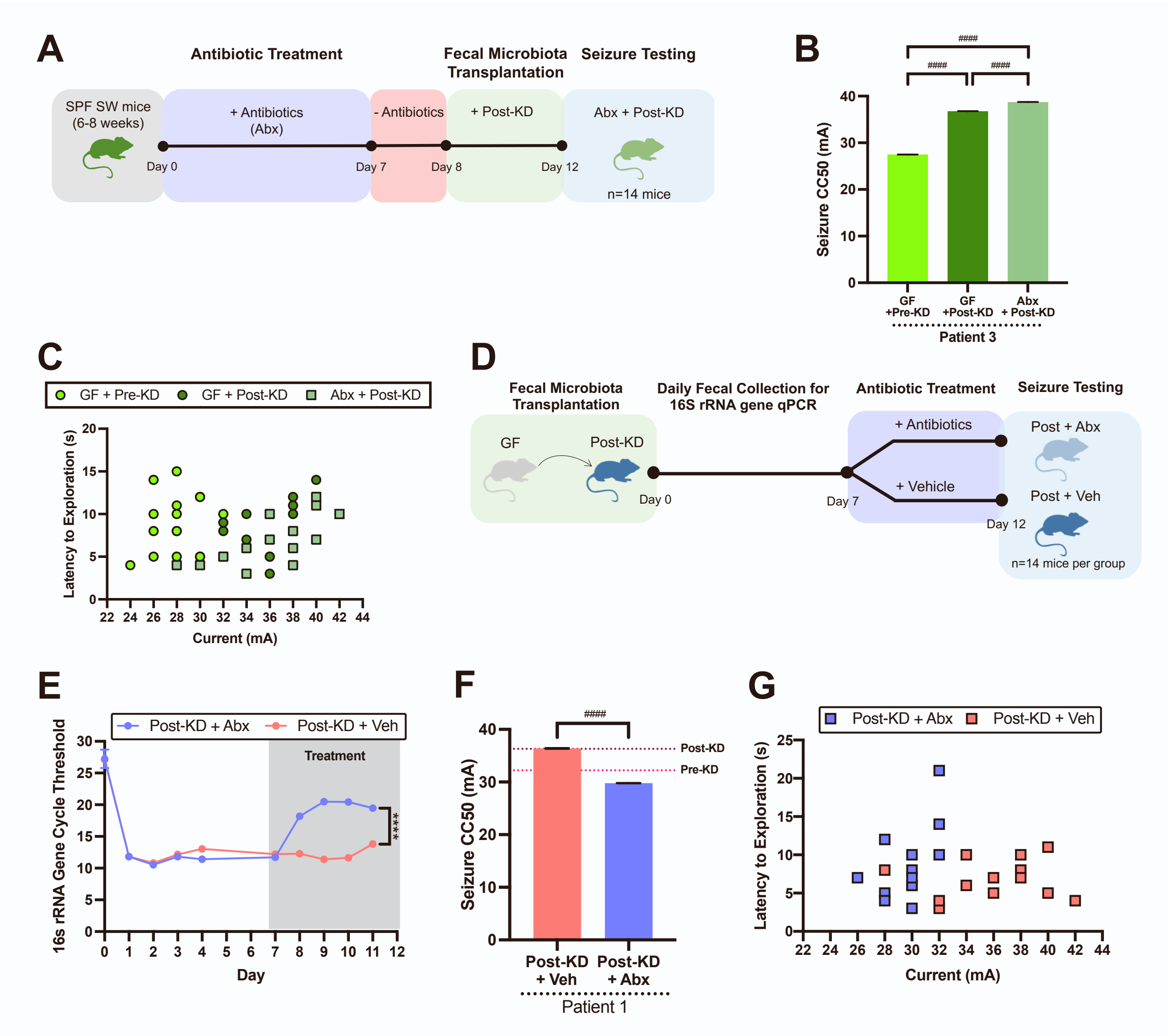

Figure S2: Antibiotic treatment abrogates the seizure protective effects of inoculation with the clinical KD-associated human gut microbiome, Related to Figure 1. **(A)** Experimental schematic for transfer of human donor fecal microbiota sample into conventionally colonized (SPF) mice pre-treated with oral antibiotic (Abx) for 7 days, followed by 6-Hz psychomotor seizure testing. **(B)** 6-Hz seizure thresholds for Abx-treated mice inoculated with patient 3 post-KD human microbiota (n=14) compared to GF mice inoculated with patient 3 post-KD (n=14) and patient 3 pre-KD (n=14) relative control fecal microbiota from Figure 1B. (One-way ANOVA with Tukey's, with # denoting statistical differences when considering within-patient recipient mice as technical replicates). **(C)** Latency to exploration for inoculated Abx-treated (n=14) vs inoculated GF mice from Figure 1B. **(D)** Experimental schematic for transfer of human donor fecal microbiota samples to germ-free (GF) mice, followed by 5 days of oral Abx or vehicle (Veh) treatment, and then 6-Hz psychomotor seizure testing. **(E)** Bacterial loads as measured by quantitative PCR of the 16S rRNA gene from fecal pellets collected once daily before and during Abx or Veh treatment (two-way ANOVA with Sidak, n=3 cages of 3 mice each). **(F)** 6-Hz seizure thresholds for mice inoculated with patient 1 post-KD human microbiota treated with Abx (n=12) or Veh (n=14). Reference lines denote seizure thresholds for mice inoculated with patient 1 post-KD (n=14) and pre-KD (n=14) relative control fecal microbiota from Figure 1B (One-way ANOVA with Tukey's, with # denoting statistical differences when considering within-patient recipient mice as technical

replicates). **(G)** Latency to exploration for each Abx (n=12) and Veh (n=14) mouse that underwent 6-Hz psychomotor seizure testing. Data is displayed as mean ± SEM, unless otherwise noted. (One-way ANOVA with Tukey's, with # denoting statistical differences when considering within-patient recipient mice as technical replicates) $^{****}p < 0.0001$. $^{\#\#\#\#}p < 0.0001$ (for within-patient mouse recipients); SPF, specific pathogen free; SW, Swiss Webster; Abx, antibiotics; KD, ketogenic diet; GF, germ free; Veh, vehicle.

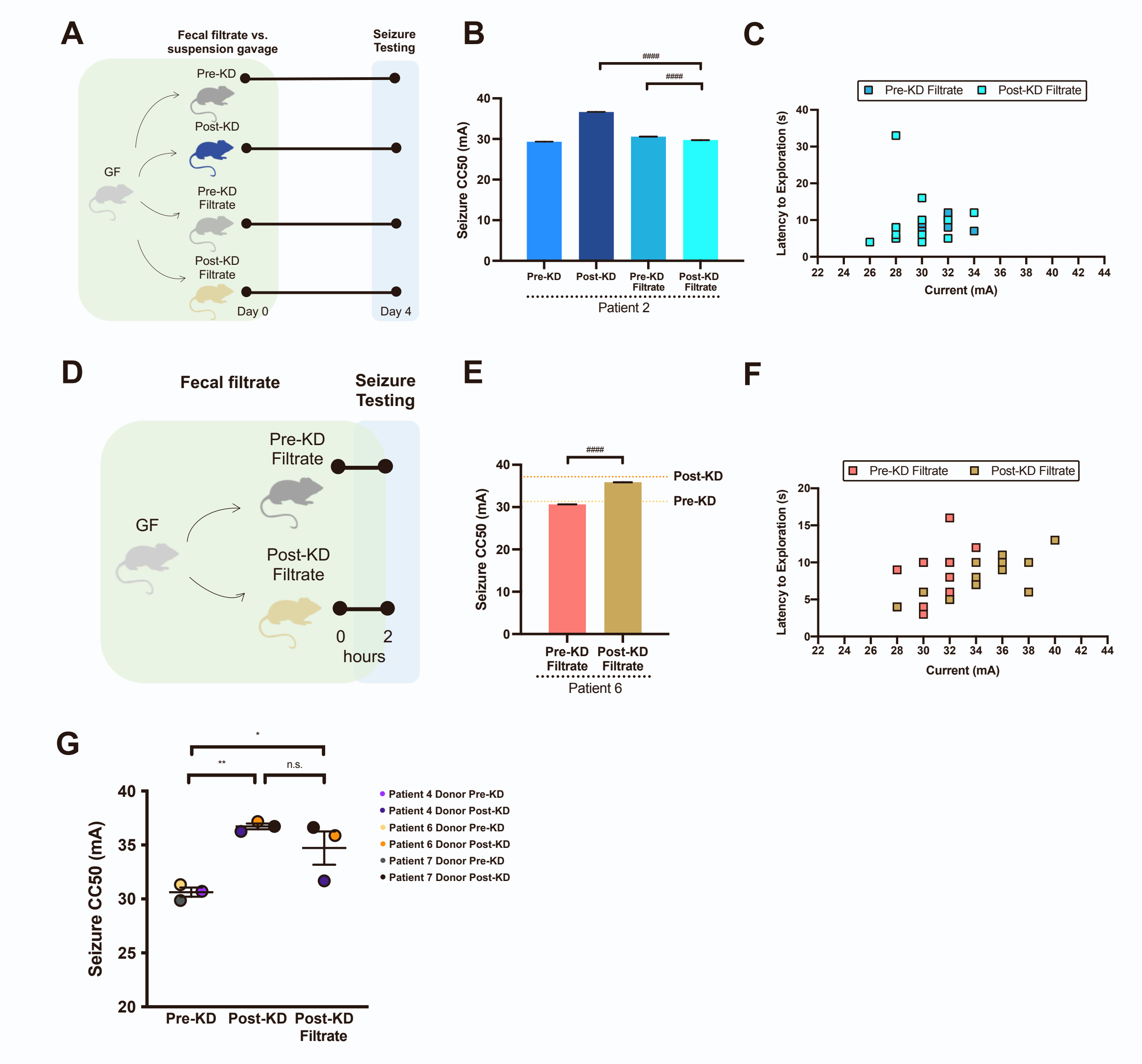

Figure S3: Sterile filtration prevents the seizure protective effects of transfer of the clinical KD-associated human gut microbiome, Related to Figure 1. **(A)** Experimental design for administration of human donor fecal filtrate samples to germ-free (GF) mice, followed by 6-Hz seizure testing 4 days later. **(B)** Seizure thresholds for mice treated with sterile filtered patient 2 pre-KD (n=14) filtrate and sterile filtered patient 2 post-KD (n=13) filtrate fecal samples compared to seizure thresholds for mice transplanted with unfiltered patient 2 post-KD (n=14) and unfiltered patient 2 pre-KD (n=14) relative control fecal microbiota from Figure 1B (One-way ANOVA with Tukey's, with # denoting statistical differences when considering within-patient recipient mice as technical replicates). **(C)** Latency to exploration for mice treated with sterile filtered pre-KD (n=14) and sterile filtered post-KD (n=13) that underwent 6-Hz psychomotor seizure testing. **(D)** Experimental design for administration of human donor fecal filtrate samples to germ-free (GF) mice, followed by 6-Hz seizure testing 2 hours later. **(E)** Seizure thresholds for mice treated with sterile filtered patient 6 pre-KD filtrate (n=13) and sterile filtered patient 6 post-KD filtrate (n=14) fecal samples. Reference lines denote seizure thresholds for mice transplanted with unfiltered patient 6 post-KD (n=14) and unfiltered patient 6 pre-KD (n=14) relative control fecal microbiota from Figure 1B. (One-way ANOVA with Tukey's, with # denoting statistical differences when considering within-patient recipient mice as technical replicates). **(F)** Latency to exploration for mice treated with sterile filtered pre-KD (n=13) and sterile filtered post-KD (n=14) that underwent

6-Hz psychomotor seizure testing. **(G)** Seizure threshold comparisons for mouse cohorts colonized with pre-KD or post-KD human fecal microbiota or treated with sterile filtered post-KD filtrate from Patients 4, 6, and 7. (One-way ANOVA with Tukey's). Data is displayed as mean ± SEM, unless otherwise noted. *$p<0.05$, **$p<0.01$, n.s = no statistical significance; ####$p <0.0001$ (for within-patient mouse recipients); KD, ketogenic diet.

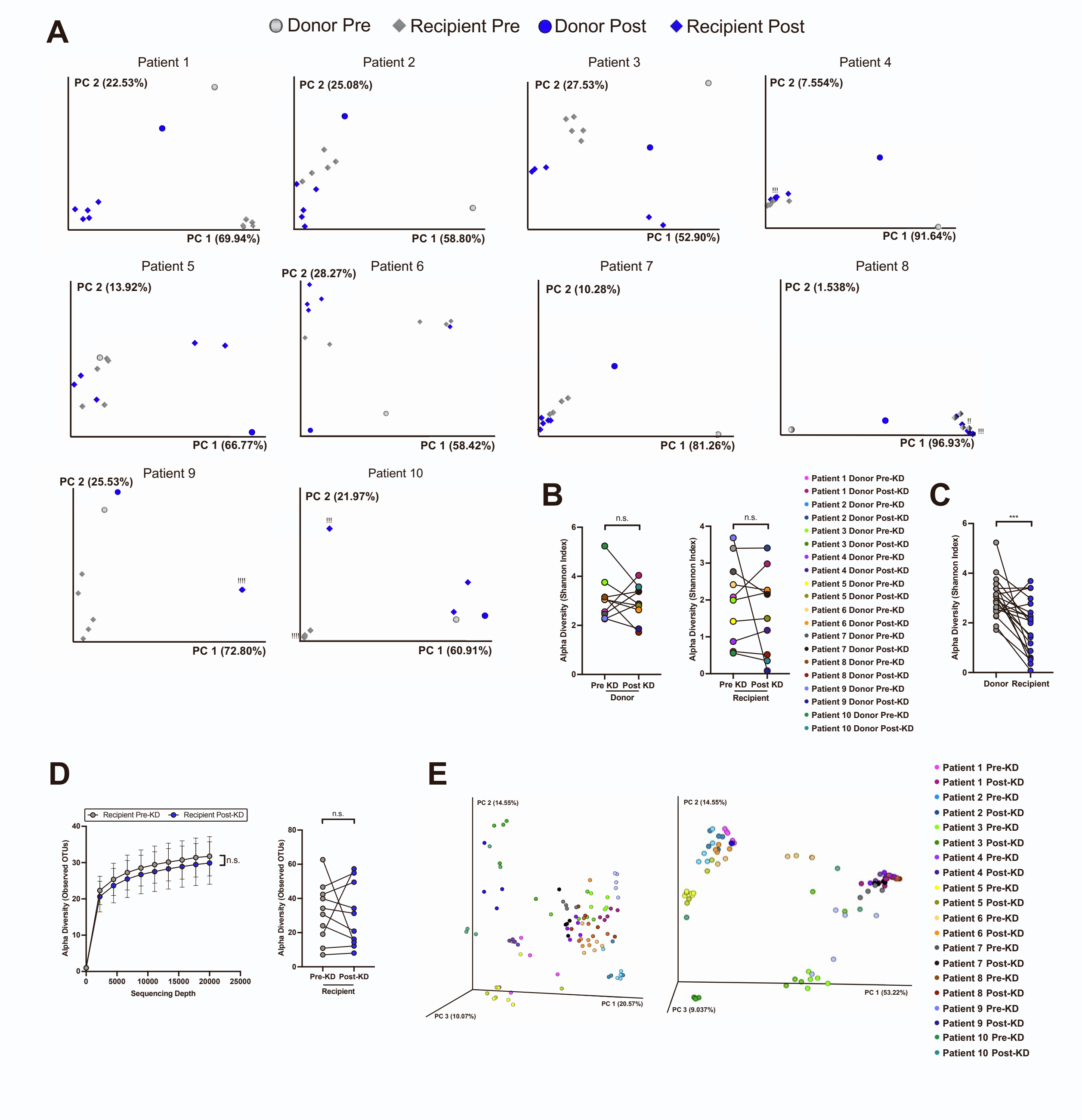

Figure S4: Taxonomic fidelity of human microbiota transfer to mice, Related to Figure 1. **(A)** Principal coordinates analysis of weighted UniFrac distances from 16S rRNA gene sequencing of fecal samples from matched human donors and mouse recipients (for each graph: n = 1 donor patient (10 patients total), 4-5 recipient cages of recipient mice per pre-KD vs. post-KD condition, ! = 1 overlapping data point not visible). **(B)** Shannon index alpha-diversity of fecal microbiota from human donor pre-KD and post-KD samples (left) and matched mouse recipient pre-KD and post-KD samples (right) (two-tailed, Wilcoxon matched-pairs signed rank test, donors: n=10 patients, recipients: n=10 per patient condition, where each n is an average from 4-5 cages per patient). **(C)** Shannon index alpha-diversity of fecal microbiota from all human donor samples (n=20 patients) and all matched mouse recipient samples (two-tailed, Wilcoxon matched-pairs signed rank test; n=20 patient conditions, where each n is an average from 4-5 cages per patient). **(D)** Alpha diversity as measured by rarefaction curve (left)

and observed OTUs (right) of mouse recipient pre-KD (n=10, where each sample is the average of 4-5 recipient mice per donor patient sample) and post-KD (n=10, where each sample is the average of 4-5 recipient mice per donor patient sample) fecal microbiota samples showing no difference in alpha-diversity. (two-way ANOVA with Sidak (left); two-tailed, Wilcoxon matched-pairs signed rank test (right)). **(E)** Principal coordinate analysis of unweighted (left) and weighted (right) UniFrac distances from 16S rRNA gene sequencing of per donor mouse recipient pre-KD (n=4-5) and post-KD (n=4-5) fecal microbiota samples shifting composition when introduced to the clinical KD. Data is displayed as mean ± SEM, unless otherwise noted. ***p < 0.001, n.s.=not statistically significant; KD, ketogenic diet.

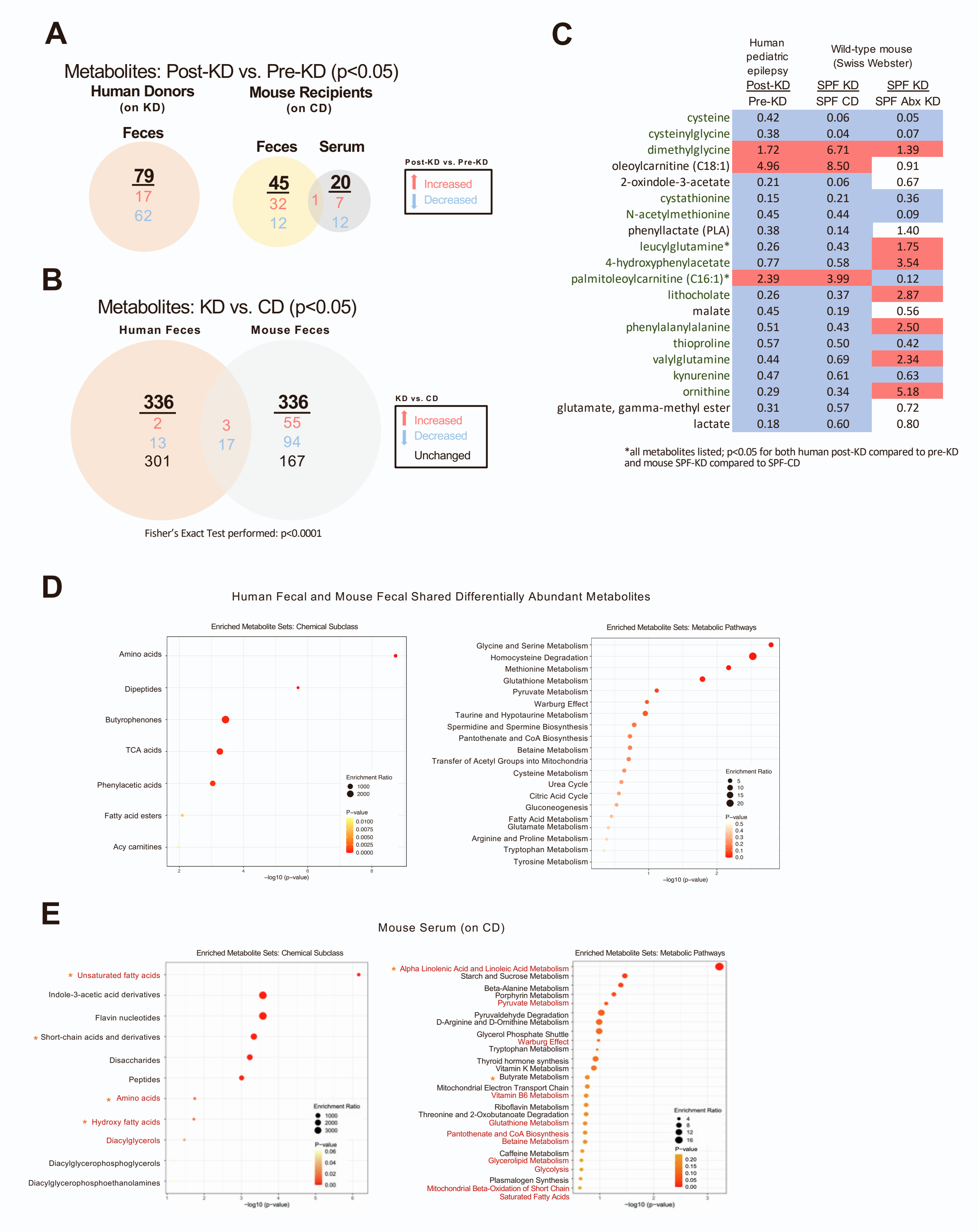

	Human pediatric epilepsy	Wild-type mouse (Swiss Webster)	
	Post-KD / Pre-KD	SPF KD / SPF CD	SPF KD / SPF Abx KD
cysteine	0.42	0.06	0.05
cysteinylglycine	0.38	0.04	0.07
dimethylglycine	1.72	6.71	1.39
oleoylcarnitine (C18:1)	4.96	8.50	0.91
2-oxindole-3-acetate	0.21	0.06	0.67
cystathionine	0.15	0.21	0.36
N-acetylmethionine	0.45	0.44	0.09
phenyllactate (PLA)	0.38	0.14	1.40
leucylglutamine*	0.26	0.43	1.75
4-hydroxyphenylacetate	0.77	0.58	3.54
palmitoleoylcarnitine (C16:1)*	2.39	3.99	0.12
lithocholate	0.26	0.37	2.87
malate	0.45	0.19	0.56
phenylalanylalanine	0.51	0.43	2.50
thioproline	0.57	0.50	0.42
valylglutamine	0.44	0.69	2.34
kynurenine	0.47	0.61	0.63
ornithine	0.29	0.34	5.18
glutamate, gamma-methyl ester	0.31	0.57	0.72
lactate	0.18	0.60	0.80

Figure S5: The clinical KD alters metabolomic profiles in human fecal samples and in fecal and serum samples of mice inoculated with human microbiota, Related to Figure 2. (A) Differentially abundant metabolites ($p<0.05$) in post-KD compared to pre-KD samples of human donor feces, mouse recipient feces, and mouse recipient serum (Two-tailed matched pairs Student's t-test, n=10 per condition, where each recipient sample is pooled from 5 recipient mice per donor patient sample) **(B)** Differentially abundant metabolites ($p<0.05$) in human feces (post-

KD compared to pre-KD) and feces of mice fed KD vs. CD chow for 14 days (Human fecal: Two-tailed matched pairs Student's t-test, n=10 per condition, where each recipient sample is pooled from 5 recipient mice per donor patient sample; Mouse fecal: two-way ANOVA with contrasts, n=8 per condition; Fisher's Exact Test). **(C)** Differentially abundant metabolites ($p<0.05$) in post-KD compared to pre-KD samples of human donor feces, which were also significantly altered in conventional mice (SPF) fed KD chow or vitamin- and mineral- matched control diet (CD) for 14 days. Red font denotes the subset of metabolites that were further altered by pre-treating KD chow-fed mice with antibiotics (Abx) to deplete gut bacteria. (human: Two-tailed matched pairs Student's t-test, n=10 per condition; mouse: ANOVA contrasts, n=8 per condition). (**D**) Metabolite set enrichment analysis of chemical subclass for the 20 differentially abundant metabolites ($p<0.05$, matched pairs Student's t-test) found in both human post-KD vs pre-KD fecal samples and SFP mouse KD vs CD fecal samples (left) (human: n=10 per condition, where each sample is pooled from 5 recipient mice per donor patient sample; mouse: n=8 per condition). Metabolite set enrichment analysis of SMPDB pathways for the 20 differentially abundant metabolites ($p<0.05$, matched pairs Student's t-test) found in both human post-KD vs pre-KD fecal samples and SFP mouse KD vs CD fecal samples (right) (human: n=10 per condition, where each sample is pooled from 5 recipient mice per donor patient sample; mouse: n=8 per condition). **(E)** Metabolite set enrichment analysis of chemical subclass for differentially abundant metabolites ($p<0.05$, matched pairs Student's t-test) in recipient mouse post-KD vs pre-KD serum samples (left) (n=10 per condition, where each sample is pooled from 5 recipient mice per donor patient sample). Metabolite set enrichment analysis of SMPDB pathways for differentially abundant metabolites ($p<0.05$, matched pairs Student's t-test) in recipient mouse post-KD vs pre-KD serum samples (right) (n=10 per condition, where each sample is pooled from 5 recipient mice per donor patient sample). Red font denotes metabolic pathways altered in post-KD vs pre-KD mouse serum that are shared with those differentially regulated in post-KD vs pre-KD mouse feces and/or human feces. Orange asterisks (*) denote additional chemical subclasses that are relevant to KD based on existing literature. Data is displayed as mean ± SEM, unless otherwise noted. KD, ketogenic diet; SPF, specific pathogen free; CD, control diet; Abx, antibiotics; SMPDB, The Small Molecule Pathway Database.

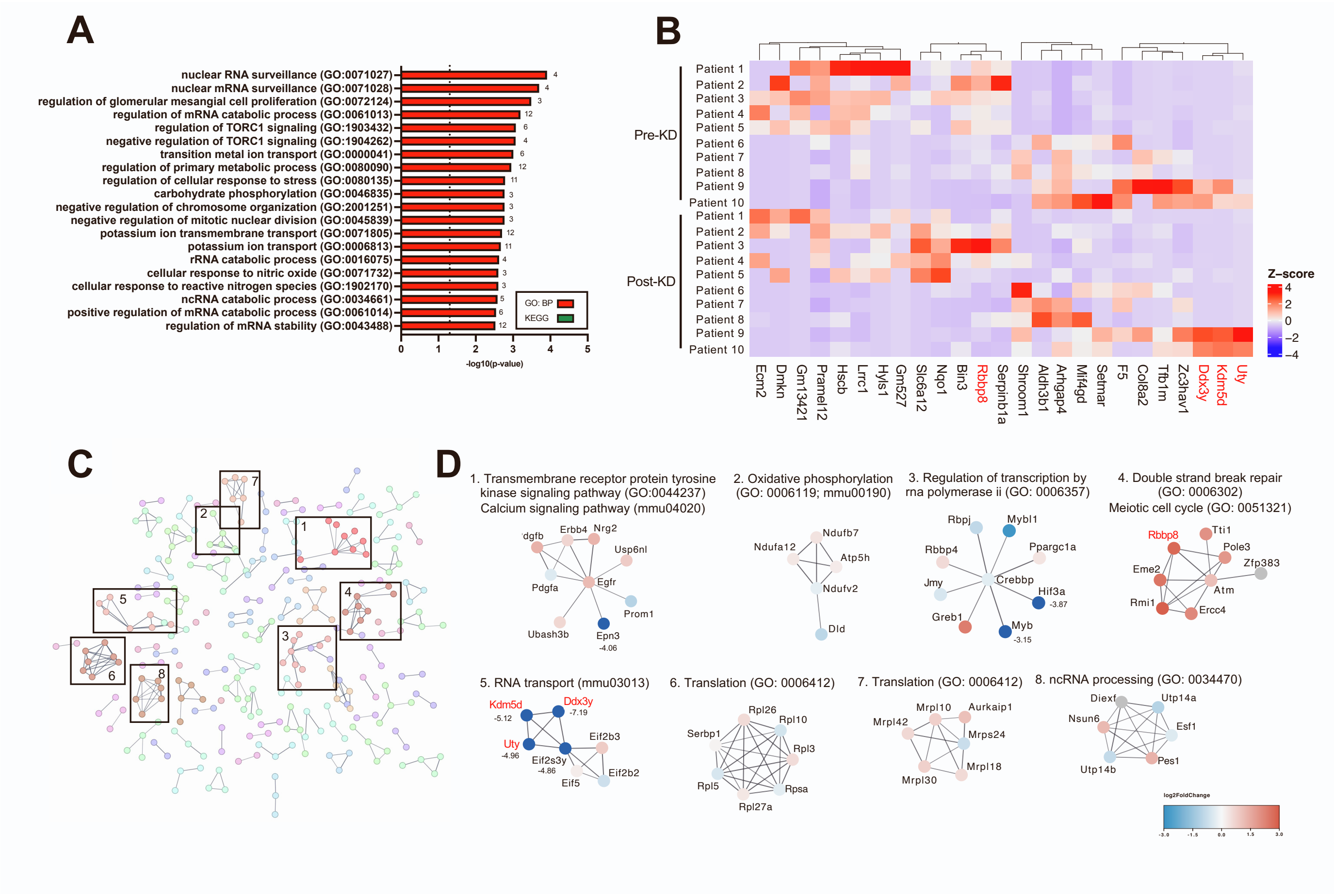

Figure S6: Mice inoculated with the post-KD microbiota exhibit alterations in the frontal cortical transcriptome, Related to Figure 3. **(A)** GO: Biological Process gene ontology of differentially expressed genes (p<0.05) in recipient mouse post-KD compared to pre-KD frontal cortex samples, top 20 ranked by p-value (n=10 per patient diet condition, where each sample is pooled from 6 recipient mice per donor patient sample). **(B)** Heatmap of top 25 differentially expressed genes in recipient mouse post-KD compared to pre-KD frontal cortex ranked by p-value, smallest to largest, with log2-fold change >2 (n=10 per patient diet condition, where each sample is pooled from 6 recipient mice per donor patient sample). **(C)** Protein interaction network with MCL clustering based upon mouse recipient post-KD and pre-KD frontal cortex transcriptomics which appeared in both GO and STRING network enrichment analyses, STRING network enrichment score >0.7 (n=10 per patient diet condition, where each sample is pooled from 6 recipient mice per donor patient sample). **(D)** Functional enrichment of top MCL sub-network clusters from frontal cortex transcriptomics STRING network analysis, proteins are colored based on their overall log2FC. If log2FC >3 or <-3, the value is listed next to the node name (n=10 per patient diet condition, where each sample is pooled from 6 recipient mice per donor patient sample). KD, ketogenic diet; GO, gene ontology; MCL, Markov Cluster Algorithm.
